# Multi-criteria decision making based on induced generalized interval neutrosophic Choquet integral

**DOI:** 10.1371/journal.pone.0242449

**Published:** 2020-12-01

**Authors:** Yangyang Jiao, Lu Wang, Jianxia Liu, Gang Ma

**Affiliations:** 1 School of Economics and Management, Wuhan University, Wuhan, China; 2 School of Management Science and Engineering, Shanxi University of Finance and Economics, Taiyuan, China; 3 School of Information Management, Nanjing University, Nanjing, China; University of Defence, SERBIA

## Abstract

In this paper, two new aggregation operators based on Choquet integral, namely the induced generalized interval neutrosophic Choquet integral average operator(IGINCIA) and the induced generalized interval neutrosophic Choquet integral geometric operator(IG-INCIG), are proposed for multi-criteria decision making problems (MCDM). Firstly, the criteria are dependent to each other and the evaluation information of the criteria are expressed by interval neutrosophic numbers. Moreover, two indices which are inspired by the geometrical structure are established to compare the interval neutrosophic numbers. Then, a MCDM method is proposed based on the proposed aggregation operators and ranking indices to cope with MCDM with interactive criteria. Lastly, an investment decision making problem is provided to illustrate the practicality and effectiveness of the proposed approach. The validity and advantages of the proposed method are analyzed by comparing with some existing approaches. By a numerical example in company investment to expand business though five alternatives with considering four criteria, the optimal decision is made.

## Introduction

Decision making problems play a very important role in our daily life. For example, consumers choose what they need from a wide variety of brands, companies make investment decisions from numerous investment projects, government makes plans of industrial resource assignment. Many researchers have been pay attentions to the methods and rules to solve the problems. With the deepening of the study, scholars found that there are a large amount of occasions which decision makers do not know the precise decision-making information. Zadeh [1] proposed the concept of fuzzy sets (FSs) which promote the development of decision theory [2]. Fuzzy sets describe the decision information with a membership degree and a non-membership degree which evaluate the possibility of the event happen and don’t happen, respectively. However, Fuzzy sets are unable to describe the uncertainty. The intuitionistic fuzzy sets (IFSs) put forward by Atanassov [3] characterized this situation properly. Then the intuitionistic fuzzy sets theory has made considerable development, such as the arising of distance measure [4], correlation coefficient [5], entropy [6], cross-entropy [7] and outranking relations [8], among others. The concept of interval-valued intuitionistic fuzzy sets is presented as well, where the membership degree and the non-membership degree are subintervals included in interval [0, 1] [9]. Various decision making methods have been proposed to solve the decision making problems with intuitionistic fuzzy information [10–14] and interval-valued intuitionistic fuzzy information [15–19]. Moreover, another development of the IFSs is the specialization of membership degree, non-membership degree and the degree of hesitation, such as the triangular fuzzy number and the trapezoidal fuzzy number. Some methods have been put forward to deal with MCDM with triangular fuzzy information [20, 21] and the trapezoidal fuzzy information [22, 23].

For an IFS, the sum of the membership degree, non-membership and hesitancy degree of a generis element in the universe equals to one, which fails to cope with the incomplete, indeterminate, and inconsistent decision information. Therefore, the concept of neutrosophic sets (NSs) [24] arose at the historic moment. The NS is a set that each element in the universe has a membership degree of truth, indeterminacy and falsity which lies in the nonstandard unit interval [0−, 1+] respectively. The concept of similarity and entropy of neutrosophic sets were proposed in [25, 26]. However, without specific description, NSs are difficult to apply to real-life situations. Therefore, single-valued neutrosophic sets (SVNSs) [27–29] and simplified neutrosophic sets (SNSs) [30] were proposed, which are the specialization of NSs. Since then, scholars did researches for MCDM problems with single-valued neutrosophic information [31–33] and simplified neutrosophic information [30, 34]. Similar to interval intuitionistic fuzzy sets, Wang et al. [35] and Chen et al. [36] proposed the concept of interval neutrosophic sets (INSs) and provided the set-theoretic operators of INSs. Besides, other methods have been used to solve MCDM problem. Shao et al. [37] adopted probabilistic neutrosophic fuzzy choquet aggregation Operators Operators to solve multi-attribute decision-making. Yörükoğlu and Aydın [38] used neutrosophic TOPSIS method to make smart container evaluation. Then a verity of methods proposed successively to solve MCDM with interval neutrosophic information, such as the extended Topsis method [39], methods based on the similarity measure [40], cross-entropy [41], or improved weighted correlation coefficient [42] and the outranking approach [43], among others. In addition, many areas have been studied, like selection of a location [44], evaluation of providers [45], evaluation of website [46, 47], e-commerce Development strategies analysis [33] and so on.

Further, some scholars considered the MCDM problems with interval neutrosophic information and interactive criteria, and proposed some valuable operators [48]. Among them are the Choquet integral operator proposed by Sun et al. [49] and generalized interval neutrosophic Choquet aggregation operators presented by Li et al. [50]. Dong et al. [51] further developed the generalized Choquet integral operator and generalized hybrid Choquet (TAIF-GHC) integral operator. Moreover, the Choquet integral operators and their generalized operators have showed great power to deal with the situation where the criteria are interactive [52, 53]. However, existing operators based on Choquet integrals are still scattered and cannot be unified into the same system. In order to extend and supplement such studies and make decision process more flexible, this paper presents two new aggregation operators based on Choquet integral, namely the induced generalized interval neutrosophic Choquet integral average operator(IG-INCIA) and the induced generalized interval neutrosophic Choquet integral geometric operator(IG-INCIG) and use them to aggregate interval neutrosophic information in MCDM with interactive criteria.

In addition, how to sort the aggregated INNs is also crucial to the MCDM with interval neutrosophic information. The methods in the current literature are mainly divided into two categories. One is to sort INNs by indicators or methods such as distance measure, similarity measure or Topsis method. The other is to establish sorting functions or order relations such as score function [54], order relations ≤_*H*_, ≤_*L*_ [49] and ≤_*P*_ [55]. Different methods have their own advantages in dealing with strict inequality properly, but almost all methods have limitations in handle equality effectively. This paper presents two indices based on geometrical structure to cope with strict inequality relationship, and one index to deal with equality relationship, which are inspired by [49].

**Fig 1:**
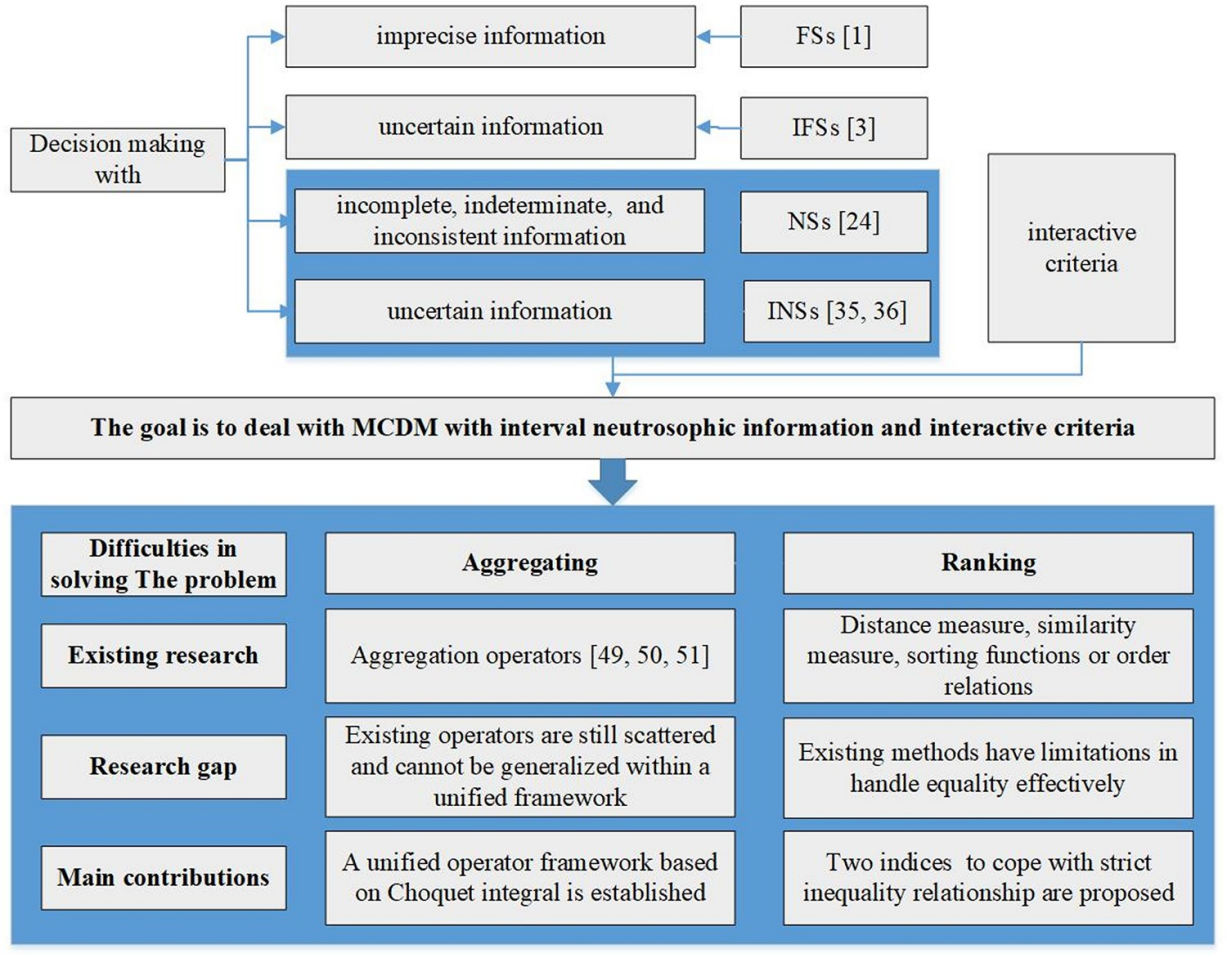
The framework of our motivation.

To show motivation of our research, the framework is shown as Fig 1. And to solve the equality problem effectively through two indices of geometrical structure and equality relationship, we proposed two new aggregation operators. Meanwhile, the indices can cope with inequality relationship. Our contributions are as follows: (1) Two new aggregation operators based on Choquet integral are presented, including induced generalized interval neutrosophic Choquet integral average operator(IG-INCIA) and the induced generalized in-terval neutrosophic Choquet integral geometric operator(IG-INCIG), which is used to aggregate interval neutrosophic information in MCDM. (2) Two indices based on geometrical structure to cope with strict inequality relationship are proposed, moreover one of them can deal with equality relationship, which extends such studies and makes decision process more flexible.

The rest of this paper is organized as follows. In Section 2, some basic concepts related to INSs and Choquet integral are briefly reviewed. Two interval neutrosophic aggregation operators based on Choquet integral are defined in Section 3. Some properties of IG-INCIA and IG-INCIG are discussed as well. Section 4 introduces two ranking indices based on the geometrical structure. Then we propose a MCDM method in section 5 to deal with the MCDM problems with interval neutrosophic information and interactive attributes. In Section 6, an illustrative example for selecting an investment place and a comparison analysis are presented to verify the effectiveness of the proposed approach. Finally, the conclusions are drawn in Section 7.

## Preliminaries

This section consists of two parts. Firstly, some basic definitions related to INSs are introduced, so as the operational laws of INSs. Secondly, the properties of Choquet integral and induced Choquet integral are presented. These preliminary knowledge will be utilised in the latter analysis.

### NS, SNS, INS and operations for INNs

**Definition 1** [24] Let X be a space of points (objects) with a generic element *x* in X. A neutrosophic set (NS) A in X is characterised by a truth-membership function *T*_*A*_(*x*), an indeterminacy-membership function *I*_*A*_(*x*) and a falsity-membership function *F*_*A*_(*x*). *T*_*A*_(*x*), *I*_*A*_(*x*) and *F*_*A*_(*x*) are real standard or non-standard subsets of ]0−, 1+ [; that is, *T*_*A*_(*x*):*X* → ]0−, 1+ [, *I*_*A*_(*x*):*X* → ]0−, 1+ [ and *F*_*A*_(*x*):*X* → ]0−, 1+ [. There is no restriction on the sum of *T*_*A*_(*x*), *I*_*A*_(*x*) and *F*_*A*_(*x*), thus 0 − ≤*supT*_*A*_(*x*) + *supI*_*A*_(*x*) + *supF*_*A*_(*x*)≤3+.

To overcome the difficulty of applying NSs to practical problems, Ye [31] reduced NSs of nonstandard intervals to a kind of simplified neutrosophic set (SNSs) of standard intervals.

**Definition 2** [31] Let a NS A in X be characterised by *T*_*A*_(*x*), *I*_*A*_(*x*) and *F*_*A*_(*x*) which are single subsets in the real standard interval [0, 1]; that is, *T*_*A*_(*x*):*X* → [0, 1], *I*_*A*_(*x*):*X* → [0, 1], and *F*_*A*_(*x*):*X* → [0, 1]. The so called simplified neutrosophic set (SNS) is denoted by *A* = {(*x*, *T*_*A*_(*x*), *I*_*A*_(*x*), *F*_*A*_(*x*))|*x* ∈ *X*}, which is a subclass of NSs. The sum of *T*_*A*_(*x*), *I*_*A*_(*x*) and *F*_*A*_(*x*) satisfies the condition 0 ≤ *T*_*A*_(*x*) + *I*_*A*_(*x*) + *F*_*A*_(*x*)≤3. If ∥*X*∥ = 1, a SNS degenerate to a simplified neutrosophic number (SNN).

Similar to interval-valued intuitionistic fuzzy set, Wang et al. [35] proposed the concept of interval neutrosophic set (INS).

**Definition 3** [35] Let X be a space of points (objects) with generic elements x in X. An interval neutrosophic set (INS) A in X is characterized by a truth-membership function *T*_*A*_(*x*), an indeterminacy-membership function *I*_*A*_(*x*), and a falsity-membership function *F*_*A*_(*x*). For each point *x* in X, $T_{A}\left(x\right)=\left[T_{A}^{L}\left(x\right),T_{A}^{U}\left(x\right)\right]\subseteq \left[0,1\right],I_{A}\left(x\right)=\left[I_{A}^{L}\left(x\right),I_{A}^{U}\left(x\right)\right]\subseteq \left[0,1\right],F_{A}\left(x\right)=\left[F_{A}^{L}\left(x\right),F_{A}^{U}\left(x\right)\right]\subseteq \left[0,1\right]$. In particular, an INS reduce to a SNS, if $T_{A}^{L}=T_{A}^{U}$, $I_{A}^{L}=I_{A}^{U}$ and $F_{A}^{L}=F_{A}^{U}$. In addition, if ∥*X*∥ = 1, an INS degenerate to an interval neutrosophic number (INN).

**Definition 4** An INS A is contained in the other INS B (*A* ⊆ *B*) if and only if $T_{A}^{L}\left(x\right)\leq T_{B}^{L}\left(x\right),T_{A}^{U}\left(x\right)\leq T_{B}^{U}\left(x\right),I_{A}^{L}\left(x\right)\geq I_{B}^{U}\left(x\right),I_{A}^{U}\left(x\right)\geq I_{B}^{U}\left(x\right),F_{A}^{L}\left(x\right)\geq F_{B}^{L}\left(x\right)$ and $F_{A}^{U}\left(x\right)\geq F_{B}^{U}\left(x\right)$ for any *x* in X [35].

Following are some operations of INNs [35, 36].

**Definition 5** Let *a*, *b* be two INNs and λ be a real number where $a=\langle \left[T_{a}^{L},T_{a}^{U}\right],\left[I_{a}^{L},I_{a}^{U}\right],\left[F_{a}^{L},F_{a}^{U}\right]\rangle$, $b=\langle \left[T_{b}^{L},T_{b}^{U}\right],\left[I_{b}^{L},I_{b}^{U}\right],\left[F_{b}^{L},F_{b}^{U}\right]\rangle$ and λ > 0. The operations for the INNs are defined as follow [35, 36].

It’s easy to prove that *a*⊕*b*, *a*⊗*b*, λ*a* and *a*^λ^ are INNs according to the Definition 2.3. The operations of INNs have some properties described in proposition 2.1.

**Proposition 1** Let *a*, *b* and *c* be three INNs and λ, λ_1_ and λ_2_ be three positive numbers. The following equations are true [35, 36].

### Fuzzy measure and induced Choquet intergal

**Definition 6** [56] A fuzzy measure on power set of *X* is a set function *μ*: *P*(*X*)→[0, 1] which satisfies the conditions below.

1. *μ*(∅) = 0, *μ*(*X*) = 1.
2. If *A*, *B* ∈ *P*(*X*) and *A* ⊆ *B*, then *μ*(*A*)≤*μ*(*B*).

In an actual decision-making process, only a finite number of situations can arise which indicate that the set *X* is finite. Thus the λ fuzzy measure is utilised to substitute the general fuzzy measure to reduce the computational complexity.

**Definition 7** [56] A fuzzy measure *μ* on *P*(*X*) is a λ fuzzy measure if for any *A*, *B* ∈ *P*(*X*), *A* ∩ *B* = ∅, the following condition is satisfied:

$$\begin{array}{c} \mu (A\cup B)=\mu (A)+\mu (B)+\lambda \cdot \mu (A)\cdot \mu (B),\lambda \in (-1,\infty ). \end{array}$$ (1)

The parameter λ = 0 indicates that *A* and *B* are independent and λ fuzzy measure is additive; λ ≠ 0 indicates that *A* and *B* have interaction, thus λ fuzzy measure is nonadditive. If λ > 0, *A* and *B* have complementary relationship, otherwise if −1 < λ < 0, *A* and *B* have redundant relationship. Three cases above describe the interactions between attributes in real decision-making problems.

Let *X* = {*x*_1_, *x*_2_, ⋯, *x*_*n*_} be a finite set where for any *i*, *j*, *i* ≠ *j*, *x*_*i*_ ∩ *x*_*j*_ = ∅. The set *X* can be expressed as $X=\overset{n}{\underset{i=1}{\cup }}x_{i}$. Then λ fuzzy measure *μ* on *P*(*X*) satisfy the following equation.

$$\begin{array}{c} \mu \left(X\right)=\mu \left(\overset{n}{\underset{i=1}{\cup }}x_{i}\right)=\{\begin{array}{cc} \frac{1}{\lambda }(\prod_{i=1}^{n}(1+\lambda \cdot \mu (x_{i}))-1), & \lambda \neq 0,\\ \sum_{i=1}^{n}\mu \left(x_{i}\right), & \lambda =0. \end{array} \end{array}$$ (2)

Due to *μ*(*X*) = 1, the value of parameter λ is determined by the equation below [56].

$$\begin{array}{c} \lambda +1=\prod_{i=1}^{n}(1+\lambda \cdot \mu (x_{i})). \end{array}$$ (3)

For any element *x*_*i*_ ∈ *X*, fuzzy measure *μ*(*x*_*i*_) represents the importance of *x*_*i*_. Similarly, for any subset *A* ⊆ *X*, fuzzy measure *μ*(*A*) represent the importance of set *A*. Both the weight and association of single element and subset can be expressed by fuzzy measure. The equation *μ*(*x*_*i*_) = 0 indicates that *x*_*i*_ is unimportant and *μ*(*T*∪*x*_*i*_)>*μ*(*x*_*i*_) indicates that *x*_*i*_ is important. The Shapley value proposed by Shapley [57] describe the importance of *x*_*i*_ relative to fuzzy measure *μ*. A basic characteristic of Shapley value of a element *x*_*i*_ is that $\sum_{i=1}^{n}\sigma \left(x_{i},\mu \right)=1.$ The equation *σ*(*x*_*i*_, *μ*) = *μ*(*x*_*i*_) holds if fuzzy measure *μ* is additive, otherwise *σ*(*x*_*i*_, *μ*)≠*μ*(*x*_*i*_).

**Definition 8** [58] Let *X* be a nonempty set, and *X* = {*x*_1_, *x*_2_, ⋯, *x*_*n*_}. *f*(*x*) be a nonnegative real-valued function on *X* and *μ* be a fuzzy measure on *P*(*X*). For *n* tuples (〈*u*_1_, *f*(*x*_1_)〉, 〈*u*_2_, *f*(*x*_2_)〉, ⋯, 〈*u*_*n*_, *f*(*x*_*n*_)〉), the function *IC*_*μ*_: (*R*^+^, *R*^+^)→*R*^+^ is called an induced Choquet integral induced by *u*_1_, *u*_2_, ⋯, *u*_*n*_ if the following equation holds.

$$\begin{array}{c} \begin{array}{c} IC_{\mu }\left(\left\langle u_{1},f\left(x_{1}\right)\right\rangle ,\left\langle u_{2},f\left(x_{2}\right)\right\rangle ,\cdots ,\left\langle u_{n},f\left(x_{n}\right)\right\rangle \right)=\\ \sum_{i=1}^{n}(\mu \left(X_{\left(i\right)}\right)-\mu \left(X_{\left(i+1\right)}\right))f\left(x_{\left(i\right)}\right), \end{array} \end{array}$$ (4)

where (*i*): {1, 2, ⋯, *n*}→{1, 2, ⋯, *n*} is a permutation on *X* such that *u*_(1)_ ≤ *u*_(2)_ ≤ ⋯ ≤ *u*_(*n*)_, *f*(*x*_(*i*)_) is the second component of the tuple 〈*u*_(*i*)_, *f*(*x*_(*i*)_)〉 and *X*_(*i*)_ = {*x*_(*i*)_, …, *x*_(*n*)_}, *X*_(*n*+1)_ = ∅. Within the pairs〈*u*_*i*_, *f*(*x*_*i*_)〉, *u*_*i*_ is called the order inducing value and *f*(*x*_*i*_) is called the argument value.

## Interval neutrosophic aggregation operators based on Choquet integral

### Induced generalized interval neutrosophic Choquet integral average operator

**Definition 9** Let *X* be a set of INNs, and *X* = {*x*_1_, *x*_2_, ⋯, *x*_*n*_}. *μ* be a fuzzy measure on *P*(*X*). *u*_*i*_ is the number determined by *x*_*i*_ and *μ*, *i* = 1, 2, ⋯, *n*. The Induced generalized interval neutrosophic Choquet integral average operator (IG-INCIA) is defined as

$$\begin{array}{c} \begin{array}{c} IG-INCIA_{\lambda }(\left\langle u_{1},x_{1}\right\rangle ,\left\langle u_{2},x_{2}\right\rangle ,\cdots ,\left\langle u_{n},x_{n}\right\rangle )={\left(\overset{n}{\underset{i=1}{⊕}}(\mu \left(X_{\left(i\right)}\right)-\mu \left(X_{\left(i+1\right)}\right))x_{\left(i\right)}^{\lambda }\right)}^{\frac{1}{\lambda }}, \end{array} \end{array}$$ (5)

where λ ∈ (0, + ∞), ((1), (2), ⋯, (*n*)) is a permutation of (1, 2, ⋯, *n*) such that *u*_(1)_ ≤ *u*_(2)_ ≤ ⋯ ≤ *u*_(*n*)_, *x*_(*i*)_ is the second component of the tuple 〈*u*_(*i*)_, *x*_(*i*)_〉 and *X*_(*i*)_ = {*x*_(*i*)_, …, *x*_(*n*)_}, *X*_(*n*+1)_ = ∅.

**Theorem 1** Let *X* be a set of INNs and *X* = {*x*_1_, *x*_2_, ⋯, *x*_*n*_} where $x_{i}=\langle \left[T_{x_{i}}^{L},T_{x_{i}}^{U}\right],\left[I_{x_{i}}^{L},I_{x_{i}}^{U}\right],\left[F_{x_{i}}^{L},F_{x_{i}}^{U}\right]\rangle$. *μ* and *u*_*i*_(*i* = 1, 2, ⋯, *n*) are the same as in definition 3.1. Then the value aggregated by the IG-INCIA operator is an INN, and

$$\begin{array}{c} IG-INCIA_{\lambda }(\left\langle u_{1},x_{1}\right\rangle ,\left\langle u_{2},x_{2}\right\rangle ,\cdots ,\left\langle u_{n},x_{n}\right\rangle )=\\ \langle [{\left(1-\prod_{i=1}^{n}{\left(1-{\left(T_{x_{\left(i\right)}}^{L}\right)}^{\lambda }\right)}^{\mu \left(X_{\left(i\right)}\right)-\mu \left(X_{\left(i+1\right)}\right)}\right)}^{\frac{1}{\lambda }},{\left(1-\prod_{i=1}^{n}{\left(1-{\left(T_{x_{\left(i\right)}}^{U}\right)}^{\lambda }\right)}^{\mu \left(X_{\left(i\right)}\right)-\mu \left(X_{\left(i+1\right)}\right)}\right)}^{\frac{1}{\lambda }}],\\ {}[1-{\left(1-\prod_{i=1}^{n}{\left(1-{\left(1-I_{x_{\left(i\right)}}^{L}\right)}^{\lambda }\right)}^{\mu \left(X_{\left(i\right)}\right)-\mu \left(X_{\left(i+1\right)}\right)}\right)}^{\frac{1}{\lambda }},1-{\left(1-\prod_{i=1}^{n}{\left(1-{\left(1-I_{x_{\left(i\right)}}^{U}\right)}^{\lambda }\right)}^{\mu \left(X_{\left(i\right)}\right)-\mu \left(X_{\left(i+1\right)}\right)}\right)}^{\frac{1}{\lambda }}],\\ {}[1-{\left(1-\prod_{i=1}^{n}{\left(1-{\left(1-F_{x_{\left(i\right)}}^{L}\right)}^{\lambda }\right)}^{\mu \left(X_{\left(i\right)}\right)-\mu \left(X_{\left(i+1\right)}\right)}\right)}^{\frac{1}{\lambda }},1-{\left(1-\prod_{i=1}^{n}{\left(1-{\left(1-F_{x_{\left(i\right)}}^{U}\right)}^{\lambda }\right)}^{\mu \left(X_{\left(i\right)}\right)-\mu \left(X_{\left(i+1\right)}\right)}\right)}^{\frac{1}{\lambda }}]\rangle , \end{array}$$ (6)

where λ ∈ (0, + ∞), ((1), (2), ⋯, (*n*)) is a permutation of (1, 2, ⋯, *n*) such that *u*_(1)_ ≤ *u*_(2)_ ≤ ⋯ ≤ *u*_(*n*)_, *x*_(*i*)_ is the second component of the tuple 〈*u*_(*i*)_, *x*_(*i*)_〉 and *X*_(*i*)_ = {*x*_(*i*)_, …, *x*_(*n*)_}, *X*_(*n*+1)_ = ∅

**Proof**. The first result is easily proved in line with Definition 3.1 and Definition 2.5. Eq (6) can be obtained by means of mathematical induction on *n*.

1. The equation holds when *n* = 1. For *n* = 2, according to the operation relation of INNs defined in Definition 2.5, we have
  $$\begin{array}{c} (\mu \left(X_{\left(1\right)}\right)-\mu \left(X_{\left(2\right)}\right))x_{1}^{\lambda }=\langle [1-{\left(1-{\left(T_{x_{1}}^{L}\right)}^{\lambda }\right)}^{\mu \left(X_{\left(1\right)}\right)-\mu \left(X_{\left(2\right)}\right)},1-{\left(1-{\left(T_{x_{1}}^{U}\right)}^{\lambda }\right)}^{\mu \left(X_{\left(1\right)}\right)-\mu \left(X_{\left(2\right)}\right)}],\\ {}[{\left(1-{\left(1-I_{x_{1}}^{L}\right)}^{\lambda }\right)}^{\mu \left(X_{\left(1\right)}\right)-\mu \left(X_{\left(2\right)}\right)},{\left(1-{\left(1-I_{x_{1}}^{U}\right)}^{\lambda }\right)}^{\mu \left(X_{\left(1\right)}\right)-\mu \left(X_{\left(2\right)}\right)}],\\ {}[{\left(1-{\left(1-F_{x_{1}}^{L}\right)}^{\lambda }\right)}^{\mu \left(X_{\left(1\right)}\right)-\mu \left(X_{\left(2\right)}\right)},{\left(1-{\left(1-F_{x_{1}}^{U}\right)}^{\lambda }\right)}^{\mu \left(X_{\left(1\right)}\right)-\mu \left(X_{\left(2\right)}\right)}]\rangle ;\\ \begin{array}{c} (\mu \left(X_{\left(2\right)}\right)-\mu \left(\emptyset \right))x_{2}^{\lambda }=\langle [1-{\left(1-{\left(T_{x_{2}}^{L}\right)}^{\lambda }\right)}^{\mu \left(X_{\left(2\right)}\right)-\mu \left(\emptyset \right)},1-{\left(1-{\left(T_{x_{2}}^{U}\right)}^{\lambda }\right)}^{\mu \left(X_{\left(2\right)}\right)-\mu \left(\emptyset \right)}],\\ {}[{\left(1-{\left(1-I_{x_{2}}^{L}\right)}^{\lambda }\right)}^{\mu \left(X_{\left(2\right)}\right)-\mu \left(\emptyset \right)},{\left(1-{\left(1-I_{x_{2}}^{U}\right)}^{\lambda }\right)}^{\mu \left(X_{\left(2\right)}\right)-\mu \left(\emptyset \right)}],\\ {}[{\left(1-{\left(1-F_{x_{2}}^{L}\right)}^{\lambda }\right)}^{\mu \left(X_{\left(2\right)}\right)-\mu \left(\emptyset \right)},{\left(1-{\left(1-F_{x_{2}}^{U}\right)}^{\lambda }\right)}^{\mu \left(X_{\left(2\right)}\right)-\mu \left(\emptyset \right)}]\rangle . \end{array} \end{array}$$
  $$\begin{array}{c} \begin{array}{c} IG-INCIA_{\lambda }(\left\langle u_{1},x_{1}\right\rangle ,\left\langle u_{2},x_{2}\right\rangle )=\\ \langle [{\left(1-\prod_{i=1}^{2}{\left(1-{\left(T_{x_{\left(i\right)}}^{L}\right)}^{\lambda }\right)}^{\mu \left(X_{\left(i\right)}\right)-\mu \left(X_{\left(i+1\right)}\right)}\right)}^{\frac{1}{\lambda }},{\left(1-\prod_{i=1}^{2}{\left(1-{\left(T_{x_{\left(i\right)}}^{U}\right)}^{\lambda }\right)}^{\mu \left(X_{\left(i\right)}\right)-\mu \left(X_{\left(i+1\right)}\right)}\right)}^{\frac{1}{\lambda }}],\\ {}[1-{\left(1-\prod_{i=1}^{2}{\left(1-{\left(1-I_{x_{\left(i\right)}}^{L}\right)}^{\lambda }\right)}^{\mu \left(X_{\left(i\right)}\right)-\mu \left(X_{\left(i+1\right)}\right)}\right)}^{\frac{1}{\lambda }},1-{\left(1-\prod_{i=1}^{2}{\left(1-{\left(1-I_{x_{\left(i\right)}}^{U}\right)}^{\lambda }\right)}^{\mu \left(X_{\left(i\right)}\right)-\mu \left(X_{\left(i+1\right)}\right)}\right)}^{\frac{1}{\lambda }}],\\ {}[1-{\left(1-\prod_{i=1}^{2}{\left(1-{\left(1-F_{x_{\left(i\right)}}^{L}\right)}^{\lambda }\right)}^{\mu \left(X_{\left(i\right)}\right)-\mu \left(X_{\left(i+1\right)}\right)}\right)}^{\frac{1}{\lambda }},1-{\left(1-\prod_{i=1}^{2}{\left(1-{\left(1-F_{x_{\left(i\right)}}^{U}\right)}^{\lambda }\right)}^{\mu \left(X_{\left(i\right)}\right)-\mu \left(X_{\left(i+1\right)}\right)}\right)}^{\frac{1}{\lambda }}]\rangle . \end{array} \end{array}$$
  $$\begin{array}{c} \begin{array}{c} IG-INCIA_{\lambda }(\left\langle u_{1},x_{1}\right\rangle ,\left\langle u_{2},x_{2}\right\rangle ,\cdots ,\left\langle u_{k},x_{k}\right\rangle )=\\ \langle [{\left(1-\prod_{i=1}^{k}{\left(1-{\left(T_{x_{\left(i\right)}}^{L}\right)}^{\lambda }\right)}^{\mu \left(X_{\left(i\right)}\right)-\mu \left(X_{\left(i+1\right)}\right)}\right)}^{\frac{1}{\lambda }},{\left(1-\prod_{i=1}^{k}{\left(1-{\left(T_{x_{\left(i\right)}}^{U}\right)}^{\lambda }\right)}^{\mu \left(X_{\left(i\right)}\right)-\mu \left(X_{\left(i+1\right)}\right)}\right)}^{\frac{1}{\lambda }}],\\ {}[1-{\left(1-\prod_{i=1}^{k}{\left(1-{\left(1-I_{x_{\left(i\right)}}^{L}\right)}^{\lambda }\right)}^{\mu \left(X_{\left(i\right)}\right)-\mu \left(X_{\left(i+1\right)}\right)}\right)}^{\frac{1}{\lambda }},1-{\left(1-\prod_{i=1}^{k}{\left(1-{\left(1-I_{x_{\left(i\right)}}^{U}\right)}^{\lambda }\right)}^{\mu \left(X_{\left(i\right)}\right)-\mu \left(X_{\left(i+1\right)}\right)}\right)}^{\frac{1}{\lambda }}],\\ {}[1-{\left(1-\prod_{i=1}^{k}{\left(1-{\left(1-F_{x_{\left(i\right)}}^{L}\right)}^{\lambda }\right)}^{\mu \left(X_{\left(i\right)}\right)-\mu \left(X_{\left(i+1\right)}\right)}\right)}^{\frac{1}{\lambda }},1-{\left(1-\prod_{i=1}^{k}{\left(1-{\left(1-F_{x_{\left(i\right)}}^{U}\right)}^{\lambda }\right)}^{\mu \left(X_{\left(i\right)}\right)-\mu \left(X_{\left(i+1\right)}\right)}\right)}^{\frac{1}{\lambda }}]\rangle . \end{array} \end{array}$$
  Then, the equation below is true which means that the result is proved when *n* = 2.
2. If Eq (6) holds for *n* = *k*, then
  $$\begin{array}{c} \begin{array}{c} \overset{k}{\underset{i=1}{⊕}}(\mu \left(X_{\left(i\right)}\right)-\mu \left(X_{\left(i+1\right)}\right))x_{\left(i\right)}^{\lambda }=\\ \langle [1-\prod_{i=1}^{k}{\left(1-{\left(T_{x_{\left(i\right)}}^{L}\right)}^{\lambda }\right)}^{\mu \left(X_{\left(i\right)}\right)-\mu \left(X_{\left(i+1\right)}\right)},1-\prod_{i=1}^{k}{\left(1-{\left(T_{x_{\left(i\right)}}^{U}\right)}^{\lambda }\right)}^{\mu \left(X_{\left(i\right)}\right)-\mu \left(X_{\left(i+1\right)}\right)}],\\ {}[\prod_{i=1}^{k}{\left(1-{\left(1-I_{x_{\left(i\right)}}^{L}\right)}^{\lambda }\right)}^{\mu \left(X_{\left(i\right)}\right)-\mu \left(X_{\left(i+1\right)}\right)},\prod_{i=1}^{k}{\left(1-{\left(1-I_{x_{\left(i\right)}}^{U}\right)}^{\lambda }\right)}^{\mu \left(X_{\left(i\right)}\right)-\mu \left(X_{\left(i+1\right)}\right)}],\\ {}[\prod_{i=1}^{k}{\left(1-{\left(1-F_{x_{\left(i\right)}}^{L}\right)}^{\lambda }\right)}^{\mu \left(X_{\left(i\right)}\right)-\mu \left(X_{\left(i+1\right)}\right)},\prod_{i=1}^{k}{\left(1-{\left(1-F_{x_{\left(i\right)}}^{U}\right)}^{\lambda }\right)}^{\mu \left(X_{\left(i\right)}\right)-\mu \left(X_{\left(i+1\right)}\right)}]\rangle . \end{array} \end{array}$$

In the case of *n* = *k* + 1,

$$\begin{array}{c} \begin{array}{c} IG-INCIA_{\lambda }(\left\langle u_{1},x_{1}\right\rangle ,\left\langle u_{2},x_{2}\right\rangle ,\cdots ,\left\langle u_{k},x_{k}\right\rangle ,\left\langle u_{k+1},x_{k+1}\right\rangle )=\\ {\left(\overset{k}{\underset{i=1}{⊕}}(\mu \left(X_{\left(i\right)}\right)-\mu \left(X_{\left(i+1\right)}\right))x_{\left(i\right)}^{\lambda }⊕(\mu \left(X_{\left(k+1\right)}\right)-\mu \left(\emptyset \right))x_{\left(k+1\right)}^{\lambda }\right)}^{\frac{1}{\lambda }}\\ \{\langle [1-\prod_{i=1}^{k}{\left(1-{\left(T_{x_{\left(i\right)}}^{L}\right)}^{\lambda }\right)}^{\mu \left(X_{\left(i\right)}\right)-\mu \left(X_{\left(i+1\right)}\right)},1-\prod_{i=1}^{k}{\left(1-{\left(T_{x_{\left(i\right)}}^{U}\right)}^{\lambda }\right)}^{\mu \left(X_{\left(i\right)}\right)-\mu \left(X_{\left(i+1\right)}\right)}],\\ {}[\prod_{i=1}^{k}{\left(1-{\left(1-I_{x_{\left(i\right)}}^{L}\right)}^{\lambda }\right)}^{\mu \left(X_{\left(i\right)}\right)-\mu \left(X_{\left(i+1\right)}\right)},\prod_{i=1}^{k}{\left(1-{\left(1-I_{x_{\left(i\right)}}^{U}\right)}^{\lambda }\right)}^{\mu \left(X_{\left(i\right)}\right)-\mu \left(X_{\left(i+1\right)}\right)}],\\ {}[\prod_{i=1}^{k}{\left(1-{\left(1-F_{x_{\left(i\right)}}^{L}\right)}^{\lambda }\right)}^{\mu \left(X_{\left(i\right)}\right)-\mu \left(X_{\left(i+1\right)}\right)},\prod_{i=1}^{k}{\left(1-{\left(1-F_{x_{\left(i\right)}}^{U}\right)}^{\lambda }\right)}^{\mu \left(X_{\left(i\right)}\right)-\mu \left(X_{\left(i+1\right)}\right)}]\rangle ⊕\\ \langle [1-{\left(1-{\left(T_{x_{k+1}}^{L}\right)}^{\lambda }\right)}^{\mu \left(X_{\left(k+1\right)}\right)-\mu \left(\emptyset \right)},1-{\left(1-{\left(T_{x_{k+1}}^{U}\right)}^{\lambda }\right)}^{\mu \left(X_{\left(k+1\right)}\right)-\mu \left(\emptyset \right)}],\\ {}[{\left(1-{\left(1-I_{x_{k+1}}^{L}\right)}^{\lambda }\right)}^{\mu \left(X_{\left(k+1\right)}\right)-\mu \left(\emptyset \right)},{\left(1-{\left(1-I_{x_{k+1}}^{U}\right)}^{\lambda }\right)}^{\mu \left(X_{\left(k+1\right)}\right)-\mu \left(\emptyset \right)}],\\ {\left[{\left(1-{\left(1-F_{x_{k+1}}^{L}\right)}^{\lambda }\right)}^{\mu \left(X_{\left(k+1\right)}\right)-\mu \left(\emptyset \right)},{\left(1-{\left(1-F_{x_{k}+1}^{U}\right)}^{\lambda }\right)}^{\mu \left(X_{\left(k+1\right)}\right)-\mu \left(\emptyset \right)}]\rangle \right\}}^{\frac{1}{\lambda }} \end{array} \end{array}$$

Thus the Eq (6) still holds for *n* = *k* + 1 by calculating according to the operational properties of INNs presented in Definition 2.5, as shown below.

The proof of Theorem 3.1 is complete.

$$\begin{array}{c} \begin{array}{c} IG-INCIA_{\lambda }(\left\langle u_{1},x_{1}\right\rangle ,\left\langle u_{2},x_{2}\right\rangle ,\cdots ,\left\langle u_{k},x_{k}\right\rangle ,\left\langle u_{k+1},x_{k+1}\right\rangle )=\\ \langle [{\left(1-\prod_{i=1}^{k+1}{\left(1-{\left(T_{x_{\left(i\right)}}^{L}\right)}^{\lambda }\right)}^{\mu \left(X_{\left(i\right)}\right)-\mu \left(X_{\left(i+1\right)}\right)}\right)}^{\frac{1}{\lambda }},{\left(1-\prod_{i=1}^{k+1}{\left(1-{\left(T_{x_{\left(i\right)}}^{U}\right)}^{\lambda }\right)}^{\mu \left(X_{\left(i\right)}\right)-\mu \left(X_{\left(i+1\right)}\right)}\right)}^{\frac{1}{\lambda }}],\\ {}[1-{\left(1-\prod_{i=1}^{k+1}{\left(1-{\left(1-I_{x_{\left(i\right)}}^{L}\right)}^{\lambda }\right)}^{\mu \left(X_{\left(i\right)}\right)-\mu \left(X_{\left(i+1\right)}\right)}\right)}^{\frac{1}{\lambda }},1-{\left(1-\prod_{i=1}^{k+1}{\left(1-{\left(1-I_{x_{\left(i\right)}}^{U}\right)}^{\lambda }\right)}^{\mu \left(X_{\left(i\right)}\right)-\mu \left(X_{\left(i+1\right)}\right)}\right)}^{\frac{1}{\lambda }}],\\ \left[1-{\left(1-\prod_{i=1}^{k+1}{\left(1-{\left(1-F_{x_{\left(i\right)}}^{L}\right)}^{\lambda }\right)}^{\mu \left(X_{\left(i\right)}\right)-\mu \left(X_{\left(i+1\right)}\right)}\right)}^{\frac{1}{\lambda }},1-{\left(1-\prod_{i=1}^{k+1}{\left(1-{\left(1-F_{x_{\left(i\right)}}^{U}\right)}^{\lambda }\right)}^{\mu \left(X_{\left(i\right)}\right)-\mu \left(X_{\left(i+1\right)}\right)}\right)}^{\frac{1}{\lambda }}\right]\rangle . \end{array} \end{array}$$

### Induced generalized interval neutrosophic Choquet integral geometric operator

**Definition 10** Let *X* be a set of INNs, and *X* = {*x*_1_, *x*_2_, ⋯, *x*_*n*_}. *μ* be a fuzzy measure on *P*(*X*). *u*_*i*_ is the number determined by *x*_*i*_ and *μ*, *i* = 1, 2, ⋯, *n*. The Induced generalized interval neutrosophic Choquet integral geometric operator (IG-INCIG) is defined as

$$\begin{array}{c} \begin{array}{c} IG-INCIG_{\lambda }(\left\langle u_{1},x_{1}\right\rangle ,\left\langle u_{2},x_{2}\right\rangle ,\cdots ,\left\langle u_{n},x_{n}\right\rangle )=\frac{1}{\lambda }(\overset{n}{\underset{i=1}{⊗}}{\left(\lambda x_{\left(i\right)}\right)}^{\mu \left(X_{\left(i\right)}\right)-\mu \left(X_{\left(i+1\right)}\right)}) \end{array} \end{array}$$ (7)

where λ ∈ (0, + ∞), ((1), (2), ⋯, (*n*)) is a permutation of (1, 2, ⋯, *n*) such that *u*_(1)_ ≤ *u*_(2)_ ≤ ⋯ ≤ *u*_(*n*)_, *x*_(*i*)_ is the second component of the tuple 〈*u*_(*i*)_, *x*_(*i*)_〉 and *X*_(*i*)_ = {*x*_(*i*)_, …, *x*_(*n*)_}, *X*_(*n*+1)_ = ∅.

**Theorem 2** Let *X* be a set of INNs and *X* = {*x*_1_, *x*_2_, ⋯, *x*_*n*_} where $x_{i}=\langle \left[T_{x_{i}}^{L},T_{x_{i}}^{U}\right],\left[I_{x_{i}}^{L},I_{x_{i}}^{U}\right],\left[F_{x_{i}}^{L},F_{x_{i}}^{U}\right]\rangle$. *μ* and *u*_*i*_(*i* = 1, 2, ⋯, *n*) are the same as in definition 3.2. Then the value aggregated by the IG-INCIG operator is an interval neutrosophic number, and

$$\begin{array}{c} \begin{array}{c} IG-INCIG_{\lambda }(\left\langle u_{1},x_{1}\right\rangle ,\left\langle u_{2},x_{2}\right\rangle ,\cdots ,\left\langle u_{n},x_{n}\right\rangle )=\\ \langle [1-{\left(1-\prod_{i=1}^{n}{\left(1-{\left(1-T_{x_{\left(i\right)}}^{L}\right)}^{\lambda }\right)}^{\mu \left(X_{\left(i\right)}\right)-\mu \left(X_{\left(i+1\right)}\right)}\right)}^{\frac{1}{\lambda }},1-{\left(1-\prod_{i=1}^{n}{\left(1-{\left(1-T_{x_{\left(i\right)}}^{U}\right)}^{\lambda }\right)}^{\mu \left(X_{\left(i\right)}\right)-\mu \left(X_{\left(i+1\right)}\right)}\right)}^{\frac{1}{\lambda }}],\\ {}[{\left(1-\prod_{i=1}^{n}{\left(1-{\left(I_{x_{\left(i\right)}}^{L}\right)}^{\lambda }\right)}^{\mu \left(X_{\left(i\right)}\right)-\mu \left(X_{\left(i+1\right)}\right)}\right)}^{\frac{1}{\lambda }},{\left(1-\prod_{i=1}^{n}{\left(1-{\left(I_{x_{\left(i\right)}}^{U}\right)}^{\lambda }\right)}^{\mu \left(X_{\left(i\right)}\right)-\mu \left(X_{\left(i+1\right)}\right)}\right)}^{\frac{1}{\lambda }}],\\ {}[{\left(1-\prod_{i=1}^{n}{\left(1-{\left(F_{x_{\left(i\right)}}^{L}\right)}^{\lambda }\right)}^{\mu \left(X_{\left(i\right)}\right)-\mu \left(X_{\left(i+1\right)}\right)}\right)}^{\frac{1}{\lambda }},{\left(1-\prod_{i=1}^{n}{\left(1-{\left(F_{x_{\left(i\right)}}^{U}\right)}^{\lambda }\right)}^{\mu \left(X_{\left(i\right)}\right)-\mu \left(X_{\left(i+1\right)}\right)}\right)}^{\frac{1}{\lambda }}]\rangle , \end{array} \end{array}$$ (8)

where λ ∈ (0, + ∞), ((1), (2), ⋯, (*n*)) is a permutation of (1, 2, ⋯, *n*) such that *u*_(1)_ ≤ *u*_(2)_ ≤ ⋯ ≤ *u*_(*n*)_, *x*_(*i*)_ is the second component of the tuple 〈*u*_(*i*)_, *x*_(*i*)_〉 and *X*_(*i*)_ = {*x*_(*i*)_, …, *x*_(*n*)_}, *X*_(*n*+1)_ = ∅.

**Proof**. The first result is easily proved from Definition 3.2 and Definition 2.5. Eq (8) can be obtained by using mathematical induction on *n*. The proof process is similar to the proof of Theorem 3.1, thus omit here.

### Properties of IG-INCIA and IG-INCIG

The following propositions present the properties of IG-INCIA and IG-INCIG.

**Proposition 2 (Idempotency)** Let *X* be a set of INNs and *X* = {*x*_1_, *x*_2_, ⋯, *x*_*n*_}. *μ* be a fuzzy measure on *P*(*X*). If *x*_*i*_ = *x* for *i* = 1, 2, ⋯, *n*, then

$$\begin{array}{c} IG-INCIA_{\lambda }\left(\left\langle u_{1},x_{1}\right\rangle ,\left\langle u_{2},x_{2}\right\rangle ,\cdots ,\left\langle u_{n},x_{n}\right\rangle \right)=x. \end{array}$$ (9)

$$\begin{array}{c} IG-INCIG_{\lambda }\left(\left\langle u_{1},x_{1}\right\rangle ,\left\langle u_{2},x_{2}\right\rangle ,\cdots ,\left\langle u_{n},x_{n}\right\rangle \right)=x. \end{array}$$ (10)

**Proposition 3 (Monotonicity)** Let *X* and *Y* be two sets of INNs. *X* = {*x*_1_, *x*_2_, ⋯, *x*_*n*_} where $x_{i}=\langle \left[T_{x_{i}}^{L},T_{x_{i}}^{U}\right],\left[I_{x_{i}}^{L},I_{x_{i}}^{U}\right],\left[F_{x_{i}}^{L},F_{x_{i}}^{U}\right]\rangle$ and *Y* = {*y*_1_, *y*_2_, ⋯, *y*_*n*_} where $y_{i}=\langle \left[T_{y_{i}}^{L},T_{y_{i}}^{U}\right],\left[I_{y_{i}}^{L},I_{y_{i}}^{U}\right],\left[F_{y_{i}}^{L},F_{y_{i}}^{U}\right]\rangle$. *μ* be the same fuzzy measure on *P*(*X*) and *P*(*Y*). If $T_{x_{i}}^{L}\leq T_{y_{i}}^{L},T_{x_{i}}^{U}\leq T_{y_{i}}^{U},I_{x_{i}}^{L}\geq I_{y_{i}}^{L},I_{x_{i}}^{U}\geq I_{y_{i}}^{U},F_{x_{i}}^{L}\geq F_{y_{i}}^{L},F_{x_{i}}^{U}\geq F_{y_{i}}^{U},$ then

$$\begin{array}{c} \begin{array}{c} IG-INCIA_{\lambda }\left(\left\langle u_{1},x_{1}\right\rangle ,\left\langle u_{2},x_{2}\right\rangle ,\cdots ,\left\langle u_{n},x_{n}\right\rangle \right)\leq \\ IG-INCIA_{\lambda }\left(\left\langle u_{1},y_{1}\right\rangle ,\left\langle u_{2},y_{2}\right\rangle ,\cdots ,\left\langle u_{n},y_{n}\right\rangle \right), \end{array} \end{array}$$ (11)

$$\begin{array}{c} \begin{array}{c} IG-INCIG_{\lambda }\left(\left\langle u_{1},x_{1}\right\rangle ,\left\langle u_{2},x_{2}\right\rangle ,\cdots ,\left\langle u_{n},x_{n}\right\rangle \right)\leq \\ IG-INCIG_{\lambda }(\left\langle u_{1},y_{1}\right\rangle ,\left\langle u_{2},y_{2}\right\rangle ,\cdots ,\left\langle u_{n},y_{n}\right\rangle , \end{array} \end{array}$$ (12)

with respect to the same order.

**Proof**. The proof of the monotonicity of IG-INCIA operator is provided, and the same method can be used to prove the monotonicity of IG-INCIG operator. We consider the case where there are two interval neutrosophic numbers to aggregate, which are $x_{i}=\langle \left[T_{x_{i}}^{L},T_{x_{i}}^{U}\right],\left[I_{x_{i}}^{L},I_{x_{i}}^{U}\right],\left[F_{x_{i}}^{L},F_{x_{i}}^{U}\right]\rangle$ and $y_{i}=\langle \left[T_{y_{i}}^{L},T_{y_{i}}^{U}\right],\left[I_{y_{i}}^{L},I_{y_{i}}^{U}\right],\left[F_{y_{i}}^{L},F_{y_{i}}^{U}\right]\rangle$, *i* = 1, 2. The truth membership degree of *IG*−*INCIA*_λ_(〈*u*_1_, *x*_1_〉, 〈*u*_2_, *x*_2_〉) is

$$\begin{array}{c} \begin{array}{c} {}[{\left(1-{\left(1-{\left(T_{x_{\left(1\right)}}^{L}\right)}^{\lambda }\right)}^{\mu \left(X_{\left(1\right)}\right)-\mu \left(X_{\left(2\right)}\right)}{\left(1-{\left(T_{x_{\left(2\right)}}^{L}\right)}^{\lambda }\right)}^{\mu \left(X_{\left(2\right)}\right)-\mu \left(X_{\left(3\right)}\right)}\right)}^{\frac{1}{\lambda }},\\ {\left(1-{\left(1-{\left(T_{x_{\left(1\right)}}^{U}\right)}^{\lambda }\right)}^{\mu \left(X_{\left(1\right)}\right)-\mu \left(X_{\left(2\right)}\right)}{\left(1-{\left(T_{x_{\left(2\right)}}^{U}\right)}^{\lambda }\right)}^{\mu \left(X_{\left(2\right)}\right)-\mu \left(X_{\left(3\right)}\right)}\right)}^{\frac{1}{\lambda }}]. \end{array} \end{array}$$

In order to draw the conclusion, the following inequality is prove to be established firstly.

$$\begin{array}{c} {\left(1-{\left(1-{\left(T_{x_{\left(1\right)}}^{L}\right)}^{\lambda }\right)}^{\mu \left(X_{\left(1\right)}\right)-\mu \left(X_{\left(2\right)}\right)}{\left(1-{\left(T_{x_{\left(2\right)}}^{L}\right)}^{\lambda }\right)}^{\mu \left(X_{\left(2\right)}\right)-\mu \left(X_{\left(3\right)}\right)}\right)}^{\frac{1}{\lambda }}\\ \leq {\left(1-{\left(1-{\left(T_{y_{\left(1\right)}}^{L}\right)}^{\lambda }\right)}^{\mu \left(Y_{\left(1\right)}\right)-\mu \left(Y_{\left(2\right)}\right)}{\left(1-{\left(T_{y_{\left(2\right)}}^{L}\right)}^{\lambda }\right)}^{\mu \left(Y_{\left(2\right)}\right)-\mu \left(Y_{\left(3\right)}\right)}\right)}^{\frac{1}{\lambda }}. \end{array}$$

Power functions are increasing function in [0, + ∞]. Thus the inequality above equals to

$$\begin{array}{c} \begin{array}{c} {\left(1-{\left(T_{x_{\left(1\right)}}^{L}\right)}^{\lambda }\right)}^{\mu \left(X_{\left(1\right)}\right)-\mu \left(X_{\left(2\right)}\right)}{\left(1-{\left(T_{x_{\left(2\right)}}^{L}\right)}^{\lambda }\right)}^{\mu \left(X_{\left(2\right)}\right)-\mu \left(X_{\left(3\right)}\right)}\geq \\ {\left(1-{\left(T_{y_{\left(1\right)}}^{L}\right)}^{\lambda }\right)}^{\mu \left(Y_{\left(1\right)}\right)-\mu \left(Y_{\left(2\right)}\right)}{\left(1-{\left(T_{y_{\left(2\right)}}^{L}\right)}^{\lambda }\right)}^{\mu \left(Y_{\left(2\right)}\right)-\mu \left(Y_{\left(3\right)}\right)}. \end{array} \end{array}$$

Due to *μ*(*X*_(1)_)−*μ*(*X*_(2)_) = *μ*(*Y*_(1)_)−*μ*(*Y*_(2)_), *μ*(*X*_(2)_)−*μ*(*X*_(3)_) = *μ*(*Y*_(2)_)−*μ*(*Y*_(3)_), $1-{\left(T_{x_{\left(1\right)}}^{L}\right)}^{\lambda }\geq 1-{\left(T_{y_{\left(1\right)}}^{L}\right)}^{\lambda }$ and $1-{\left(T_{x_{\left(2\right)}}^{L}\right)}^{\lambda }\geq 1-{\left(T_{y_{\left(2\right)}}^{L}\right)}^{\lambda }$, the inequality holds when two interval neutrosophic numbers are aggregated. For the case where there are *n* interval neutrosophic numbers, aggregate the first two interval neutrosophic numbers and then use the aggregated interval neutrosophic number to aggregate with the third one. The rest can be done in the same manner. Thus the proposition holds for the true membership degree. The indeterminacy and falsity membership degrees can be prove in the same way.

**Proposition 4 (Boundedness)** *X* is a set of INNs and *X* = {*x*_1_, *x*_2_, ⋯, *x*_*n*_} where $x_{i}=\langle \left[T_{x_{i}}^{L},T_{x_{i}}^{U}\right],\left[I_{x_{i}}^{L},I_{x_{i}}^{U}\right],\left[F_{x_{i}}^{L},F_{x_{i}}^{U}\right]\rangle$. *μ* is a fuzzy measure on *P*(*X*).

Let

$$\begin{array}{c} x^{-}=\langle [\min_{i}T_{x_{i}}^{L},\min_{i}T_{x_{i}}^{U}],[\max_{i}I_{x_{i}}^{L},\max_{i}I_{x_{i}}^{U}],[\max F_{x_{i}}^{L},\max_{i}F_{x_{i}}^{U}]\rangle ,\\ x^{+}=\langle [\max_{i}T_{x_{i}}^{L},\max_{i}T_{x_{i}}^{U}],[\min_{i}I_{x_{i}}^{L},\min_{i}I_{x_{i}}^{U}],[\min F_{x_{i}}^{L},\min_{i}F_{x_{i}}^{U}]\rangle . \end{array}$$

Then

$$\begin{array}{c} x^{-}\leq IG-INCIA_{\lambda }\left(\left\langle u_{1},x_{1}\right\rangle ,\left\langle u_{2},x_{2}\right\rangle ,\cdots ,\left\langle u_{n},x_{n}\right\rangle \right)\leq x^{+}, \end{array}$$ (13)

$$\begin{array}{c} x^{-}\leq IG-INCIG_{\lambda }\left(\left\langle u_{1},x_{1}\right\rangle ,\left\langle u_{2},x_{2}\right\rangle ,\cdots ,\left\langle u_{n},x_{n}\right\rangle \right)\leq x^{+}. \end{array}$$ (14)

**Proof**. The boundedness can be proved according to the monotonicity of the two operators in Proposition 3.2.

**Theorem 3** When parameters take different values, IG-INCIA and IG-INCIG will degenerate to different interval neutrosophic aggregation operators, specific as follows.

1. When λ = 1, IG-INCIA degenerate to induced interval neutrosophic Choquet integral average(I-INCIA) operator, and IG-INCIG degenerate to induced interval neutrosophic Choquet integral geometric(I-INCIG) operator.
  $$\begin{array}{c} \begin{array}{c} I-INCIA(\left\langle u_{1},x_{1}\right\rangle ,\left\langle u_{2},x_{2}\right\rangle ,\cdots ,\left\langle u_{n},x_{n}\right\rangle )=\\ (\overset{n}{\underset{i=1}{⊕}}(\mu \left(X_{\left(i\right)}\right)-\mu \left(X_{\left(i+1\right)}\right))x_{\left(i\right)}). \end{array} \end{array}$$ (15)
  $$\begin{array}{c} \begin{array}{c} I-INCIG(\left\langle u_{1},x_{1}\right\rangle ,\left\langle u_{2},x_{2}\right\rangle ,\cdots ,\left\langle u_{n},x_{n}\right\rangle )=\\ \left(\overset{n}{\underset{i=1}{⊗}}{\left(x_{\left(i\right)}\right)}^{\mu \left(X_{\left(i\right)}\right)-\mu \left(X_{\left(i+1\right)}\right)}\right). \end{array} \end{array}$$ (16)
  where ((1), (2), ⋯, (*n*)) is a permutation of (1, 2, ⋯, *n*) such that *u*_(1)_ ≤ *u*_(2)_ ≤ ⋯ ≤ *u*_(*n*)_, *x*_(*i*)_ is the second component of the tuple 〈*u*_(*i*)_, *x*_(*i*)_〉 and *X*_(*i*)_ = {*x*_(*i*)_, …, *x*_(*n*)_}, *X*_(*n*+1)_ = ∅.
2. When *u*_*i*_ = *x*_*i*_, IG-INCIA degenerate to generalized interval neutrosophic Choquet integral average(G-INCIA) operator, and IG-INCIG degenerate to generalized interval neutrosophic Choquet integral geometric(G-INCIG) operator.
  $$\begin{array}{c} G-INCIA_{\lambda }(x_{1},x_{2},\cdots ,x_{n})={\left(\overset{n}{\underset{i=1}{⊕}}\left(\mu \left(X_{\left(i\right)}\right)-\mu \left(X_{\left(i+1\right)}\right)\right)x_{\left(i\right)}^{\lambda }\right)}^{\frac{1}{\lambda }}. \end{array}$$ (17)
  $$\begin{array}{c} G-INCIG_{\lambda }(x_{1},x_{2},\cdots ,x_{n})=\frac{1}{\lambda }(\overset{n}{\underset{i=1}{⊗}}{\left(\lambda x_{\left(i\right)}\right)}^{\mu \left(X_{\left(i\right)}\right)-\mu \left(X_{\left(i+1\right)}\right)}). \end{array}$$ (18)
  where λ ∈ (0, + ∞), ((1), (2), ⋯, (*n*)) is a permutation of (1, 2, ⋯, *n*) such that *x*_(1)_ ≤ *x*_(2)_ ≤ ⋯ ≤ *x*_(*n*)_ (determined by the order relationship of interval neutrosophic numbers), and *X*_(*i*)_ = {*x*_(*i*)_, …, *x*_(*n*)_}, *X*_(*n*+1)_ = ∅.
3. When *u*_*i*_ = *x*_*i*_ and λ = 1, IG-INCIA degenerate into interval neutrosophic Choquet integral average(INCIA) operator, and IG-INCIG degenerate into interval neutrosophic Choquet integral geometric(INCIG) operator.
  $$\begin{array}{c} INCIA(x_{1},x_{2},\cdots ,x_{n})=(\overset{n}{\underset{i=1}{⊕}}(\mu \left(X_{\left(i\right)}\right)-\mu \left(X_{\left(i+1\right)}\right))x_{\left(i\right)}). \end{array}$$ (19)
  $$\begin{array}{c} INCIG(x_{1},x_{2},\cdots ,x_{n})=(\overset{n}{\underset{i=1}{⊗}}{\left(x_{\left(i\right)}\right)}^{\mu \left(X_{\left(i\right)}\right)-\mu \left(X_{\left(i+1\right)}\right)}). \end{array}$$ (20)
  where ((1), (2), ⋯, (*n*)) is a permutation of (1, 2, ⋯, *n*) such that *x*_(1)_ ≤ *x*_(2)_ ≤ ⋯ ≤ *x*_(*n*)_ (determined by the order relationship of interval neutrosophic numbers), and *X*_(*i*)_ = {*x*_(*i*)_, …, *x*_(*n*)_}, *X*_(*n*+1)_ = ∅.

## Two order relation based on geometrical structure

**Fig 2:**
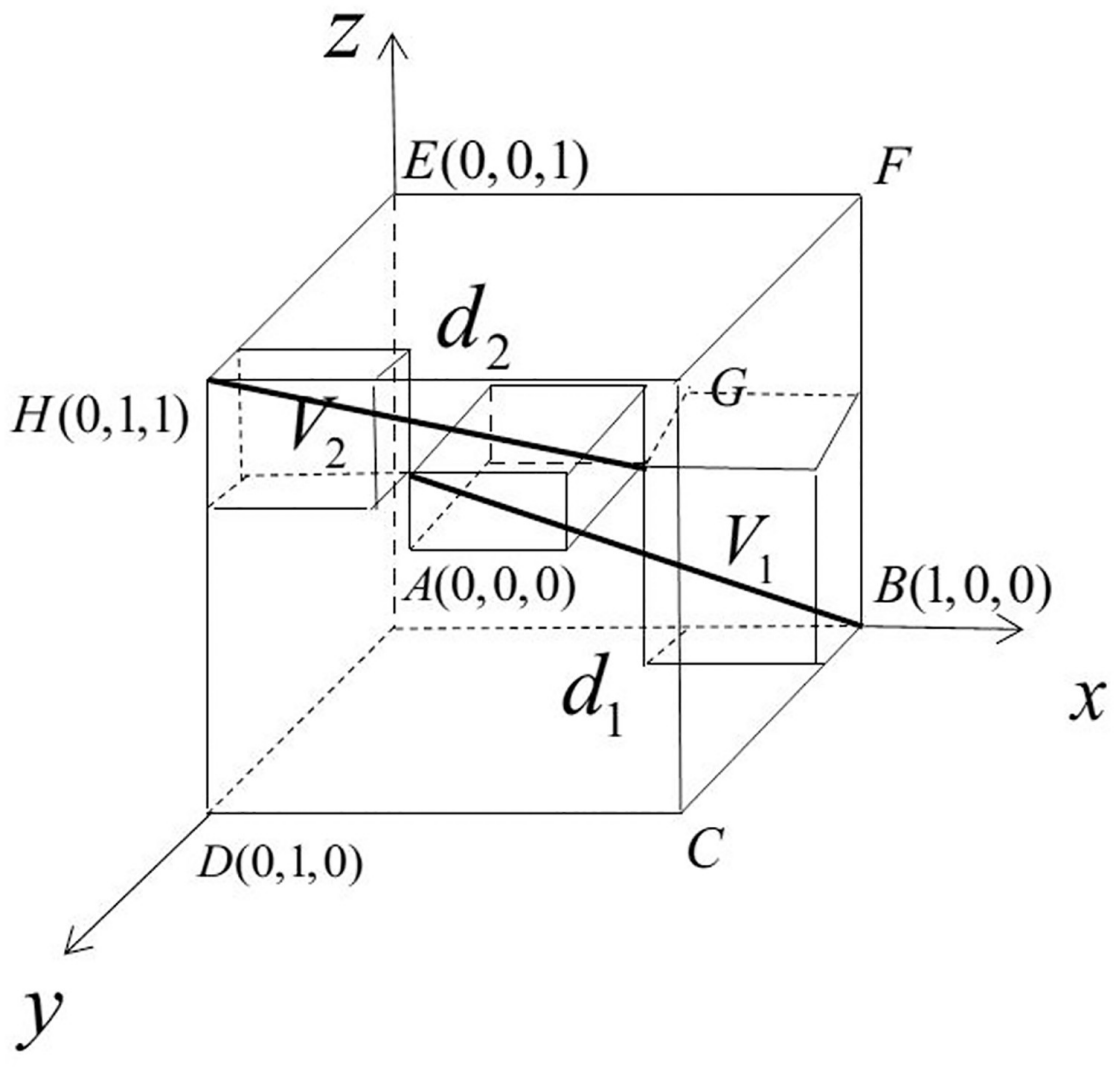
The technical neutrosophic cube.

An INN $a=\langle \left[T_{a}^{L},T_{a}^{U}\right],\left[I_{a}^{L},I_{a}^{U}\right],\left[F_{a}^{L},F_{a}^{U}\right]\rangle$ can be seen as a cube in three-dimensional space which is generated by three basis, as shown in Fig 2, where *x* is truth axis, *y* is false axis and *z* is indeterminate axis [49]. The value of three components range in the interval [0, 1], thus an INN cube is included in the unit cube in three-dimensional space which is called the technical neutrosophic cube.

The superiors point and inferiors point are *B*(1, 0, 0) and *H*(0, 1, 1) in the technical neutrosophic cube. Therefore, the shorter distance between a point *α* and *B*(1, 0, 0) is, and the longer distance between *α* and *H*(0, 1, 1) is, the bigger *α* is. Similarly, as shown in Fig 2, a cube *V* in the technical neutrosophic cube is more preference if the volume of *V*_1_ is smaller and the volume of *V*_2_ is bigger, the distance between the point $\left(T_{a}^{L},I_{a}^{U},F_{a}^{U}\right)$ and *B*(1, 0, 0), namely *d*_1_, is shorter and the distance between the point $\left(T_{a}^{U},I_{a}^{L},F_{a}^{L}\right)$ and *H*(0, 1, 1), namely *d*_2_, is longer. Based on the ideas mentioned above, a ranking index is proposed.

**Definition 11** Let *a* be an INN. $a=\langle \left[T_{a}^{L},T_{a}^{U}\right],\left[I_{a}^{L},I_{a}^{U}\right],[F_{a}^{L},F_{a}^{U}]\rangle$. A ranking index of INNs is defined as

$$\begin{array}{c} \delta \left(a\right)=\frac{V_{2}+d_{2}}{V_{1}+V_{2}+d_{1}+d_{2}}, \end{array}$$ (21)

where

$$\begin{array}{c} V_{1}=(1-T_{a}^{U})I_{a}^{L}F_{a}^{L},V_{2}=T_{a}^{L}(1-I_{a}^{U})(1-F_{a}^{U}),\\ \begin{array}{c} d_{1}=\sqrt{{\left(1-T_{a}^{L}\right)}^{2}+{\left(I_{a}^{U}\right)}^{2}+{\left(F_{a}^{U}\right)}^{2}},\\ d_{2}=\sqrt{{\left(T_{a}^{U}\right)}^{2}+{\left(1-I_{a}^{L}\right)}^{2}+{\left(1-F_{a}^{L}\right)}^{2}}. \end{array} \end{array}$$

The value of *δ*(*a*) range in [0, 1]. If *a* = 〈[1, 1], [0, 0], [0, 0]〉, then *δ*(*a*) = 1, and if *a* = 〈[0, 0], [1, 1], [1, 1]〉, then *δ*(*a*) = 0.

The following Theorem shows that the ranking index proposed is well-defined.

**Theorem 4** *Let* $a=\langle \left[T_{a}^{L},T_{a}^{U}\right],\left[I_{a}^{L},I_{a}^{U}\right],\left[F_{a}^{L},F_{a}^{U}\right]\rangle$ *and* $b=\langle \left[T_{b}^{L},T_{b}^{U}\right],\left[I_{b}^{L},I_{b}^{U}\right],\left[F_{b}^{L},F_{b}^{U}\right]\rangle$. *If* $T_{a}^{L}\leq T_{b}^{L},T_{a}^{U}\leq T_{b}^{U},I_{a}^{L}\geq I_{b}^{L},I_{a}^{U}\geq I_{b}^{U},F_{a}^{L}\geq F_{b}^{L}$ *and* $F_{a}^{U}\geq F_{b}^{U},$ *then δ*(*a*)≤*δ*(*b*).

**Proof 1** By calculating the partial derivatives, we get

$$\begin{array}{c} \frac{\partial \delta }{\partial T^{L}}=\frac{\left(1-I^{U}\right)\left(1-F^{U}\right)\left(V_{1}+d_{1}\right)+\left(V_{2}+d_{2}\right)\frac{1-T^{L}}{d_{1}}}{{\left(V_{1}+V_{2}+d_{1}+d_{2}\right)}^{2}}\geq 0,\\ \frac{\partial \delta }{\partial T^{U}}=\frac{\frac{T^{U}}{d_{2}}\left(V_{1}+d_{1}\right)+\left(V_{2}+d_{2}\right)I^{L}F^{L}}{{\left(V_{1}+V_{2}+d_{1}+d_{2}\right)}^{2}}\geq 0,\\ \frac{\partial \delta }{\partial I^{L}}=-\frac{\frac{1-I^{L}}{d_{2}}\left(V_{1}+d_{1}\right)+\left(V_{2}+d_{2}\right)\left(1-T^{U}\right)F^{L}}{{\left(V_{1}+V_{2}+d_{1}+d_{2}\right)}^{2}}\leq 0,\\ \frac{\partial \delta }{\partial I^{L}}=-\frac{\left(1-F^{U}\right)T^{L}\left(V_{1}+d_{1}\right)+\left(V_{2}+d_{2}\right)\frac{I^{U}}{d_{1}}}{{\left(V_{1}+V_{2}+d_{1}+d_{2}\right)}^{2}}\leq 0. \end{array}$$

Similarly, $\frac{\partial \delta }{\partial F^{L}}\leq 0$ and $\frac{\partial \delta }{\partial F^{U}}\leq 0$. Therefore, *δ* is an increasing function of *T*^*L*^ and *T*^*U*^, a decreasing function of *I*^*L*^, *I*^*U*^, *F*^*L*^ and *F*^*U*^. The proof is completed.

**Fig 3:**
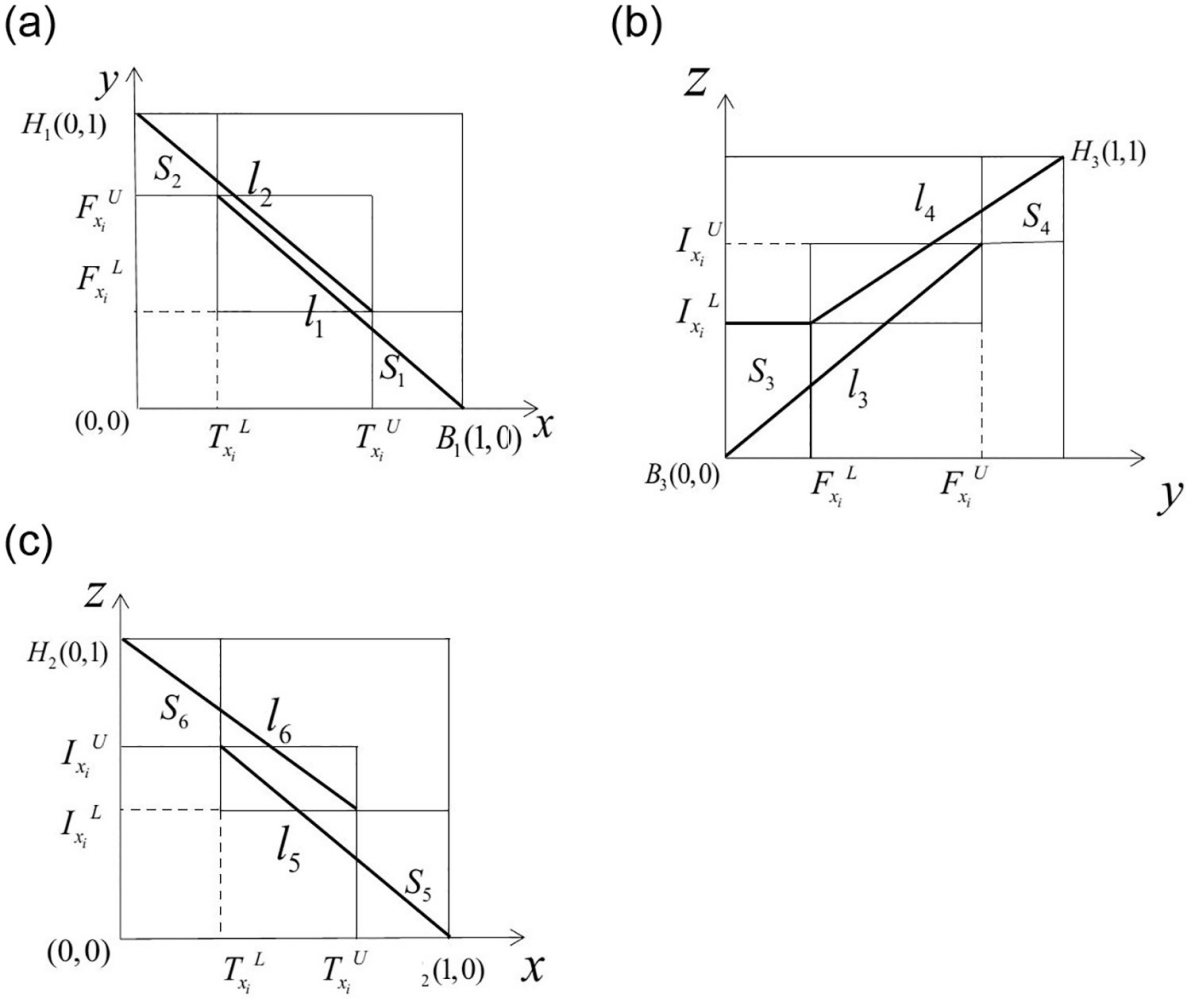
The projection of the INN cube in three coordinate planes.

Now we fous on the projection of the INN cube in three coordinate planes, as shown in Fig 3.

The subfigures of Fig 3 shows the projection of the INN cube in *x*−*y*, *y*−*z* and *x*−*z* coordinate planes. Define indices based on the projection as

$$\begin{array}{c} \delta_{1}\left(a\right)=\frac{S_{2}+l_{2}}{S_{1}+S_{2}+l_{1}+l_{2}}, \end{array}$$

where

$$\begin{array}{c} S_{1}=\left(1-T_{a}^{U}\right)F_{a}^{L},S_{2}=T_{a}^{L}\left(1-F_{a}^{U}\right),l_{1}=\sqrt{{\left(1-T_{a}^{L}\right)}^{2}+{\left(F_{a}^{U}\right)}^{2}},\\ l_{2}=\sqrt{{\left(T_{a}^{U}\right)}^{2}+{\left(1-F_{a}^{L}\right)}^{2}}.\\ \delta_{2}\left(a\right)=\frac{S_{4}+l_{4}}{S_{3}+S_{4}+l_{3}+l_{4}}, \end{array}$$

where

$$\begin{array}{c} S_{3}=I_{a}^{L}F_{a}^{L},S_{4}=\left(1-I_{a}^{U}\right)\left(1-F_{a}^{U}\right),l_{3}=\sqrt{{\left(I_{a}^{U}\right)}^{2}+{\left(F_{a}^{U}\right)}^{2}},\\ l_{4}=\sqrt{{\left(1-I_{a}^{L}\right)}^{2}+{\left(1-F_{a}^{L}\right)}^{2}}.\\ \delta_{3}\left(a\right)=\frac{S_{6}+l_{6}}{S_{5}+S_{6}+l_{5}+l_{6}}, \end{array}$$

where

$$\begin{array}{c} S_{5}=\left(1-T_{a}^{U}\right)I_{a}^{L},S_{6}=T_{a}^{L}\left(1-I_{a}^{U}\right),l_{5}=\sqrt{{\left(1-T_{a}^{L}\right)}^{2}+{\left(I_{a}^{U}\right)}^{2}},\\ l_{6}=\sqrt{{\left(T_{a}^{U}\right)}^{2}+{\left(1-I_{a}^{L}\right)}^{2}}. \end{array}$$

A ranking index obtain by weighting equally to the three indices proposed above.

**Definition 12** Let *a* be an INN. $a=\langle \left[T_{a}^{L},T_{a}^{U}\right],\left[I_{a}^{L},I_{a}^{U}\right],\left[F_{a}^{L},F_{a}^{U}\right]\rangle$. A ranking index of INNs is defined as

$$\begin{array}{c} \delta^{\prime }\left(a\right)=\frac{1}{3}\left(\delta_{1}+\delta_{2}+\delta_{3}\right) \end{array}$$ (22)

where *δ*_1_, *δ*_2_ and *δ*_3_ are three indices proposed based on projection of the INN cube.

Similar to the discussion of index *δ*, it’s easy to get that the value of *δ*′(*a*) range in [0, 1]. Furthermore, if *a* = 〈[1, 1], [0, 0], [0, 0]〉, then *δ*′(*a*) = 1, and if *a* = 〈[0, 0], [1, 1], [1, 1]〉, then *δ*′(*a*) = 0. Same method can be utilised to verify that the ranking index is well-defined.

**Definition 13** Suppose that *a* and *b* are two INNs, and *δ*(*a*) and *δ*(*b*) are ranking index of them. Then

1. If *δ*(*a*)<*δ*(*b*), then *a* is smaller than *b*, denoted by *a*<_*δ*_ *b*.
2. If *δ*(*a*) = *δ*(*b*), then *a* and *b* are indifferent to each other, denoted by *a* = _*δ*_ *b*. The order relation ≤_*δ*_ is reflexive, antisymmetric, transitive and total, and hence a total order of INNs is defined.

Same definition can be defined of the ranking index *δ*′. The order relations mentioned above describe strict inequality properly, but they have limitations in describe equality, which is shown in following example.

**Example 1** If *a* and *b* are two INNs where *a* = 〈[0.65, 0.80], [0.20, 0.452], [0.31, 0.35]〉, and *b* = 〈[0.64, 0.68], [0.25, 0.4001], [0.25, 0.35]〉. Calculated by Eq (21), we obtain that *δ*(*a*) = *δ*(*b*) = 0.6951, then *a* = _*δ*_ *b*. But it is obvious that *a* and *b* are not equal.

Considering the difficulty in describing the equality of the proposed ranking index, we propose an index to reduce the deviation. For two INNs *a* and *b*, if *a* = _*δ*_ *b*, calculate the index

$$\begin{array}{c} \gamma \left(a\right)=\left(T_{a}^{U}-T_{a}^{L}\right)\left(I_{a}^{U}-I_{a}^{L}\right)\left(F_{a}^{U}-F_{a}^{L}\right). \end{array}$$ (23)

An INN with a smaller index *γ* is more preference. The idea is inspired by geometric structure of INN cube, that is, the smaller volume of INN cube is, the better of INN is. Smaller volume indicates that the value of three components of an INN is less discrete.

## A multi-criteria decision making method based on IG-INCIA and IG-INCIG

An approach based on the IG-INCIA and IG-INCIG to solve MCDM with interval neutrosophics information and interactive criteria is presented in this section. Suppose that the MCDM problem is to choose the best alternative from *n* alternatives *X* = {*x*_1_, *x*_2_, ⋯, *x*_*n*_}. These alternatives are evaluated on *m* criteria *C* = {*c*_1_, *c*_2_, ⋯, *c*_*m*_}. Decision makers evaluate the alternative *x*_*i*_ on criterion *c*_*j*_ and give the decision information as INN *r*_*ij*_ = 〈*T*_*ij*_, *I*_*ij*_, *F*_*ij*_〉(*i* = 1, 2, ⋯, *n*;*j* = 1, 2, ⋯, *m*), where $T_{ij}=\left[t_{ij}^{L},t_{ij}^{U}\right]\subseteq \left[0,1\right]$, $I_{ij}=\left[i_{ij}^{L},i_{ij}^{U}\right]\subseteq \left[0,1\right]$ and $F_{ij}=\left[f_{ij}^{L},f_{ij}^{U}\right]\subseteq \left[0,1\right]$.

The optimal fuzzy measures *μ* on criteria set can obtain by using the Grey Relational Analysis (GRA) method [59], as shown below.

Suppose that *R* = [*r*_*ij*_]_*n*×*m*_ is the decision matrix, and $R^{+}=\left(r_{1}^{+},r_{2}^{+},r_{m}^{+}\right)$ and $R^{-}=\left(r_{1}^{-},r_{2}^{-},r_{m}^{-}\right)$ represent the positive and negative ideal alternatives respectively, where

$$\begin{array}{c} r_{j}^{+}=\left\langle \left[\max_{1\leq i\leq n}t_{ij}^{L},\max_{1\leq i\leq n}t_{ij}^{U}\right],\left[\min_{1\leq i\leq n}i_{ij}^{L},\min_{1\leq i\leq n}i_{ij}^{U}\right],\left[\min_{1\leq i\leq n}f_{ij}^{L},\min_{1\leq i\leq n}f_{ij}^{U}\right]\right\rangle ,\\ r_{j}^{-}=\left\langle \left[\min_{1\leq i\leq n}t_{ij}^{L},\min_{1\leq i\leq n}t_{ij}^{U}\right],\left[\max_{1\leq i\leq n}i_{ij}^{L},\max_{1\leq i\leq n}i_{ij}^{U}\right],\left[\max_{1\leq i\leq n}f_{ij}^{L},\max_{1\leq i\leq n}f_{ij}^{U}\right]\right\rangle . \end{array}$$

The grey relational coefficients of each alternative from the positive ideal alternative and negative alternative are determined by the following equations, respectively.

$$\begin{array}{c} \xi_{ij}^{+}=\frac{\min_{1\leq i\leq n}\min_{1\leq j\leq m}d\left(r_{ij},r_{j}^{+}\right)+\rho \max_{1\leq i\leq n}\max_{1\leq j\leq m}d\left(r_{ij},r_{j}^{+}\right)}{d\left(r_{ij},r_{j}^{+}\right)+\rho \max_{1\leq i\leq n}\max_{1\leq j\leq m}d\left(r_{ij},r_{j}^{+}\right)},\\ \xi_{ij}^{-}=\frac{\min_{1\leq i\leq n}\min_{1\leq j\leq m}d\left(r_{ij},r_{j}^{-}\right)+\rho \max_{1\leq i\leq n}\max_{1\leq j\leq m}d\left(r_{ij},r_{j}^{-}\right)}{d\left(r_{ij},r_{j}^{-}\right)+\rho \max_{1\leq i\leq n}\max_{1\leq j\leq m}d\left(r_{ij},r_{j}^{-}\right)}, \end{array}$$

for all *i* = 1, 2, ⋯, *n*;*j* = 1, 2, ⋯, *m*, where the identification coefficient *ρ* = 0.5 and the distance is Hamming distance. The non-linear programming model is constructed to obtain the optimal fuzzy measure on criteria set based on the GRA method.

$$\begin{array}{cc} \max\; & \sum_{i=1}^{n}\sum_{j=1}^{m}\frac{\xi_{ij}^{+}}{\xi_{ij}^{+}+\xi_{ij}^{-}}\sigma \left(c_{j},\mu \right)\\ \text{s.t.}\; & \mu (\emptyset )=0,\mu (C)=1,\\ & \mu (A)\leq \mu (B),\forall A,B\subseteq C,A\subseteq B,\\ & \mu \left(c_{j}\right)\in W_{c_{j}},j=1,2,\cdots ,m. \end{array}$$

where *σ*(*c*_*j*_, *μ*) is the Shapley value of the criterition *c*_*j*_(*j* = 1, 2, ⋯, *m*) about the fuzzy measure *μ*. $W_{c_{j}}$ is the rang of the attribute *c*_*j*_(*j* = 1, 2, ⋯, *m*).

**Fig 4:**
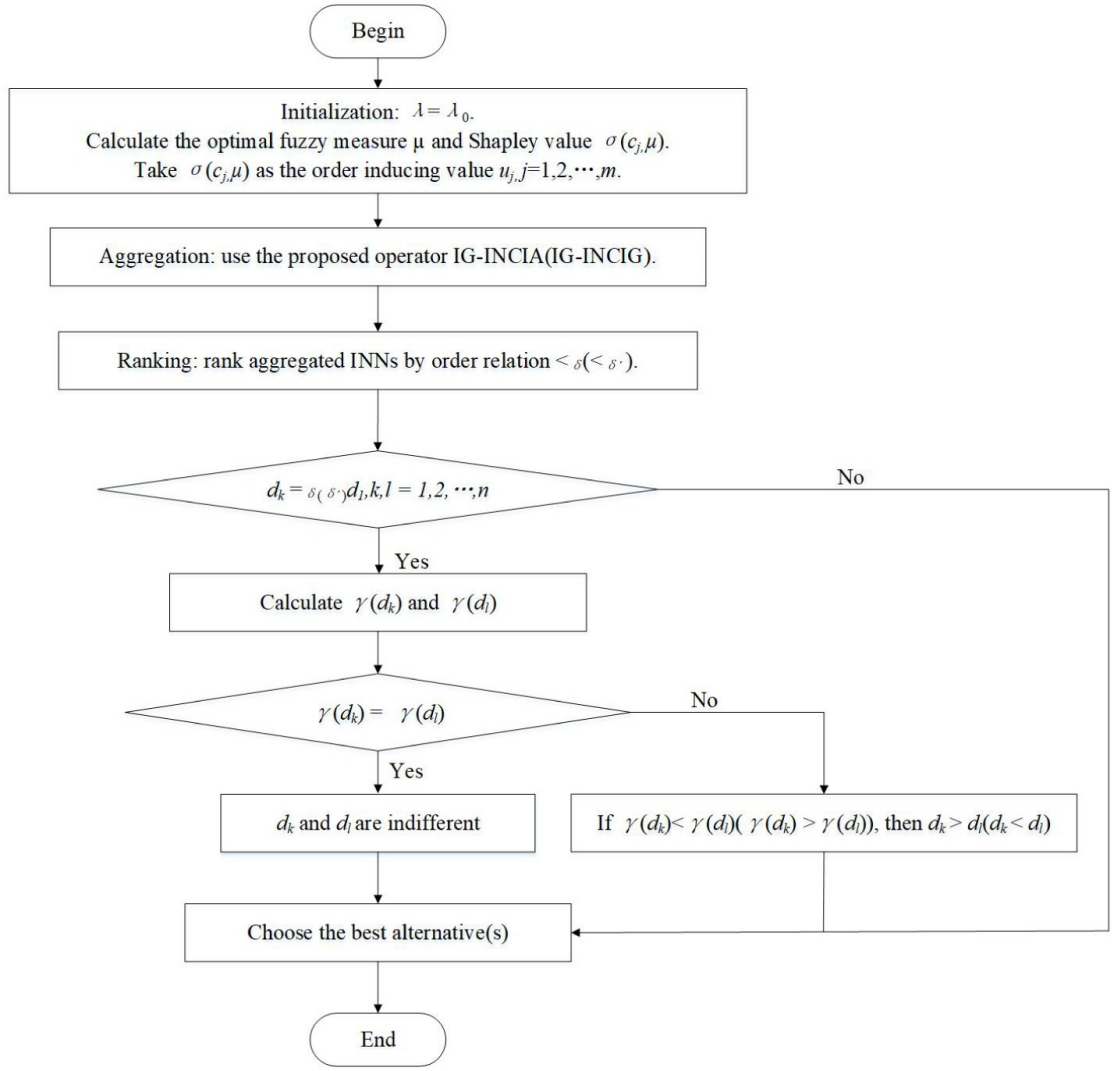
The flow chart of the proposed MCDM method.

The proposed MCDM method involves the following steps, and Fig 4 shows the flow chart of the method.

**Step 1**: Initialization. Let λ = λ_0_ be a positive constant. Calculate the optimal fuzzy measures *μ* of criteria using the method mentioned above. Take the Shapley value *σ*(*c*_*j*_, *μ*), *j* = 1, 2, ⋯, *m* as the order inducing value *u*_*j*_ of induced Choquet integral, which can be calculated by the following expression [57]
  $$\begin{array}{c} \sigma \left(c_{j},\mu \right)=\sum_{T\subseteq C\backslash c_{j}}\frac{(m-t-1)!t!}{m!}(\mu \left(T\cup c_{j}\right)-\mu \left(T\right)), \end{array}$$ (24)
  where *m* and *t* represent the cardinality of *C* and *T* respectively.
**Step 2**: Aggregation. For each alternative *x*_*i*_, aggregate the interval neutrosophics evaluation information on criteria *c*_*j*_, *j* = 1, 2, ⋯, *m*, utilizing the IG-INCIA or IG-INCIG and gain the aggregated decision information.
  $$\begin{array}{c} \begin{array}{c} d_{i}=IG-INCIA_{\lambda_{0}}(\left\langle \sigma \left(c_{1},\mu \right),d_{i1}\right\rangle ,\left\langle \sigma \left(c_{2},\mu \right),d_{i2}\right\rangle ,\\ \cdots ,\langle \sigma \left(c_{m},\mu \right),d_{im}\rangle ). \end{array} \end{array}$$ (25)
  $$\begin{array}{c} \begin{array}{c} d_{i}=IG-INCIG_{\lambda_{0}}(\left\langle \sigma \left(c_{1},\mu \right),d_{i1}\right\rangle ,\left\langle \sigma \left(c_{2},\mu \right),d_{i2}\right\rangle ,\\ \cdots ,\langle \sigma \left(c_{m},\mu \right),d_{im}\rangle ). \end{array} \end{array}$$ (26)
**Step 3**: Ranking. Calculate the ranking index *δ* or *δ*′ of *d*_*i*_, *i* = 1, 2, ⋯, *n*, and rank *n* aggregated INNs by order relation <_*δ*_ or <_*δ*′_.
  1. If there exist two aggregated INNs *d*_*k*_ and *d*_*l*_ which have the order relationship *d*_*k*_ = _*δ*(*δ*′)_ *d*_*l*_, *k*, *l* = 1, 2, ⋯, *n*, calculate *γ*(*d*_*k*_) and *γ*(*d*_*l*_).
  2. If *γ*(*d*_*k*_)<*γ*(*d*_*l*_), then *d*_*k*_ ≻ *d*_*l*_. Otherwise if *γ*(*d*_*k*_) = *γ*(*d*_*l*_), then *d*_*k*_ and *d*_*l*_ are thought to be indifferent to each other.
**Step 4**: Choose the best alternative(s).

## Numerical example analysis

For expanding its overseas business, a company is trying to choose the best foreign country (countries) from five alternatives to make an investment. The five countries are denoted by *X* = {*x*_1_, *x*_2_, *x*_3_, *x*_4_, *x*_5_}. Four main factors are selected to be criteria to make the decision, which are resources, politics and policy, economy and infrastructure, denoted by *C* = {*c*_1_, *c*_2_, *c*_3_, *c*_4_}, respectively. Ye [60] has proposed the method to convert uncertain linguistic variables, no matter cost or benefit variable, into INNs. Here we ignore the evaluation process of experts (decision makers) and the variable conversion process, and suppose the final evaluations expressed as INNs are shown in Table 1.

**Table 1:** The evaluation information.

	*c*_1_	*c*_2_
*x*_1_	〈[0.7, 0.8], [0.5, 0.7], [0.1, 0.2]〉	〈[0.6, 0.8], [0.4, 0.5], [0.3, 0.3]〉
*x*_2_	〈[0.6, 0.8], [0.4, 0.6], [0.1, 0.3]〉	〈[0.5, 0.7], [0.3, 0.5], [0.1, 0.3]〉
*x*_3_	〈[0.4, 0.6], [0.2, 0.2], [0.2, 0.4]〉	〈[0.6, 0.7], [0.4, 0.6], [0.3, 0.4]〉
*x*_4_	〈[0.4, 0.5], [0.5, 0.6], [0.4, 0.4]〉	〈[0.5, 0.6], [0.3, 0.4], [0.4, 0.5]〉
*x*_5_	〈[0.6, 0.7], [0.4, 0.5], [0.4, 0.5]〉	〈[0.8, 0.9], [0.3, 0.4], [0.1, 0.2]〉
	*c*_3_	*c*_4_
*x*_1_	〈[0.8, 0.8], [0.4, 0.6], [0.1, 0.2]〉	〈[0.7, 0.9], [0.3, 0.4], [0.2, 0.2]〉
*x*_2_	〈[0.6, 0.6], [0.2, 0.3], [0.4, 0.5]〉	〈[0.6, 0.8], [0.4, 0.4], [0.2, 0.4]〉
*x*_3_	〈[0.7, 0.8], [0.6, 0.7], [0.1, 0.2]〉	〈[0.5, 0.6], [0.5, 0.6], [0.2, 0.3]〉
*x*_4_	〈[0.6, 0.7], [0.7, 0.8], [0.2, 0.3]〉	〈[0.8, 0.9], [0.3, 0.4], [0.1, 0.2]〉
*x*_5_	〈[0.7, 0.8], [0.5, 0.6], [0.1, 0.2]〉	〈[0.5, 0.7], [0.5, 0.5], [0.2, 0.3]〉

The decision-making process is shown as follows.

**Step 1**: Initialization. Without loss of generality, we analyze the MCDM in the case of λ = 2 firstly. Other situations will be analyzed in subsequent paragraphs. The fuzzy measure of subset *A* of *C* obtains according to the above GRA-based method, as shown in Table 2. The Shapley values of criteria are *σ*(*c*_1_, *μ*) = 0.0667, *σ*(*c*_2_, *μ*) = 0.0780, *σ*(*c*_3_, *μ*) = 0.5940, *σ*(*c*_4_, *μ*) = 0.2613.
**Step 2**: Aggregation. Aggregate the INNs on four criteria utilizing the IG-INCIA and IG-INCIG respectively.
  $$\begin{array}{c} D_{i}^{IG-INCIA}={\left(d_{i}\right)}_{5\times 1}=(\begin{array}{c} \left\langle [0.7601,0.8340],[0.3751,0.5304],[0.1297,0.2062]\right\rangle \\ \left\langle [0.5987,0.6926],[0.2564,0.3495],[0.2662,0.4346]\right\rangle \\ \left\langle [0.6373,0.7436],[0.5043,0.5904],[0.1359,0.2442]\right\rangle \\ \left\langle [0.6576,0.7672],[0.4879,0.5863],[0.1821,0.2839]\right\rangle \\ \left\langle [0.6653,0.7849],[0.4709,0.5442],[0.1303,0.2347]\right\rangle \end{array}).\\ D_{i}^{IG-INCIG}={\left(d_{i}\right)}_{5\times 1}=\left(\begin{array}{c} \left\langle [0.7420,0.8205],[0.3856,0.5610],[0.1562,0.2089]\right\rangle \\ \left\langle [0.5949,0.6583],[0.2917,0.3778],[0.3303,0.4532]\right\rangle \\ \left\langle [0.5996,0.7083],[0.5475,0.6521],[0.1625,0.2675]\right\rangle \\ \left\langle [0.6084,0.7044],[0.6023,0.7077],[0.2254,0.3097]\right\rangle \\ \left\langle [0.6313,0.7666],[0.4825,0.5579],[0.1694,0.2624]\right\rangle \end{array}\right). \end{array}$$
**Step 3**: Ranking. Calculate the ranking index *δ* or *δ*′ of *d*_*i*_, *i* = 1, 2, ⋯, 5. Table 3 shows the value of *δ*(*d*_*i*_), *δ*′(*d*_*i*_) and the ranking of five alternatives.
**Step 4**: Choose the best alternatives *x*_1_ as the investment country.

**Table 2:** Subset *A* of *C* and its corresponding fuzzy measure.

*A*	*μ*(*A*)	*A*	*μ*(*A*)	*A*	*μ*(*A*)
∅	0	{*c*_1_, *c*_2_}	0.1441	{*c*_1_, *c*_2_, *c*_3_}	0.7378
{*c*_1_}	0.0664	{*c*_1_, *c*_3_}	0.6596	{*c*_1_, *c*_2_, *c*_4_}	0.4050
{*c*_2_}	0.0777	{*c*_1_, *c*_4_}	0.3271	{*c*_1_, *c*_3_, *c*_4_}	0.9217
{*c*_3_}	0.5929	{*c*_2_, *c*_3_}	0.6710	{*c*_2_, *c*_3_, *c*_4_}	0.9331
{*c*_4_}	0.2605	{*c*_2_, *c*_4_}	0.3384		
*C*	1	{*c*_3_, *c*_4_}	0.8548		

**Table 3:** The ranking of the alternatives when λ = 2.

	IG-INCIA					IG-INCIG				
	*x*_1_	*x*_2_	*x*_3_	*x*_4_	*x*_5_	*x*_1_	*x*_2_	*x*_3_	*x*_4_	*x*_5_
*δ*	0.7213	0.6669	0.6555	0.6515	0.6846	0.7020	0.6378	0.6148	0.5782	0.6610
order	*x*_1_ ≻ *x*_5_ ≻ *x*_2_ ≻ *x*_3_ ≻ *x*_4_					*x*_1_ ≻ *x*_5_ ≻ *x*_2_ ≻ *x*_3_ ≻ *x*_4_				
*δ*′	0.7137	0.6595	0.6547	0.6495	0.6766	0.6995	0.6345	0.6211	0.5880	0.6549
order	*x*_1_ ≻ *x*_5_ ≻ *x*_2_ ≻ *x*_3_ ≻ *x*_4_					*x*_1_ ≻ *x*_5_ ≻ *x*_2_ ≻ *x*_3_ ≻ *x*_4_				

The results of the case when λ = 2 indicate that the best alternative is *x*_1_ and the worst alternative is *x*_4_, no matter which aggregation operators and evaluation indices are chosen. Now we focus on the cases when λ’s value are different.

**Fig 5:**
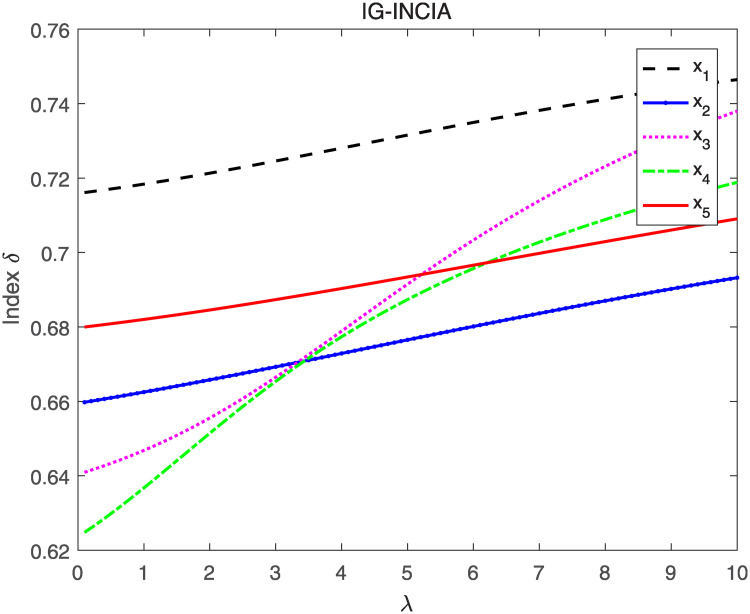
The result when IG-INCIA and index *δ* are adopted.

**Fig 6:**
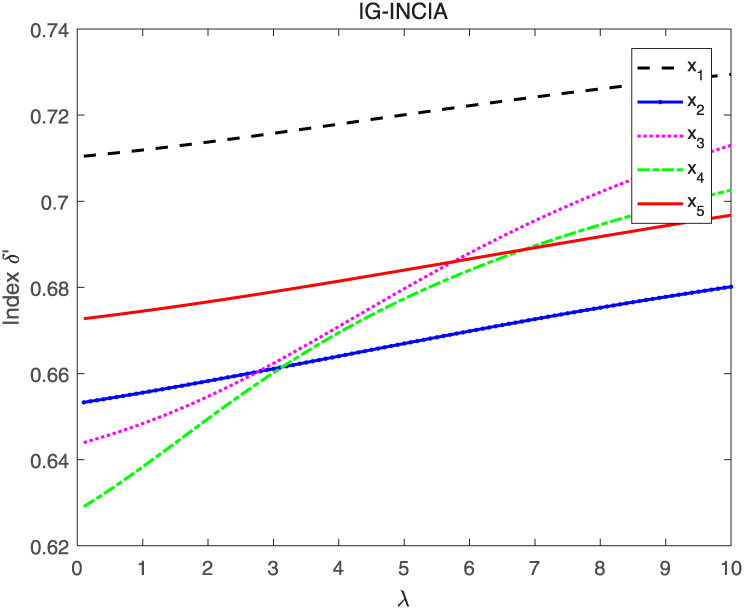
The result when IG-INCIA and index *δ*′ are adopted.

**Fig 7:**
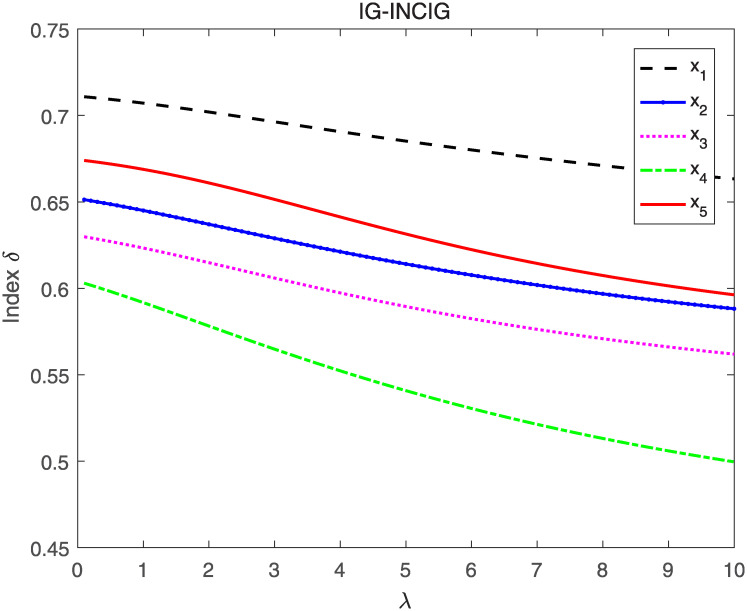
The result when IG-INCIG and index *δ* are adopted.

**Fig 8:**
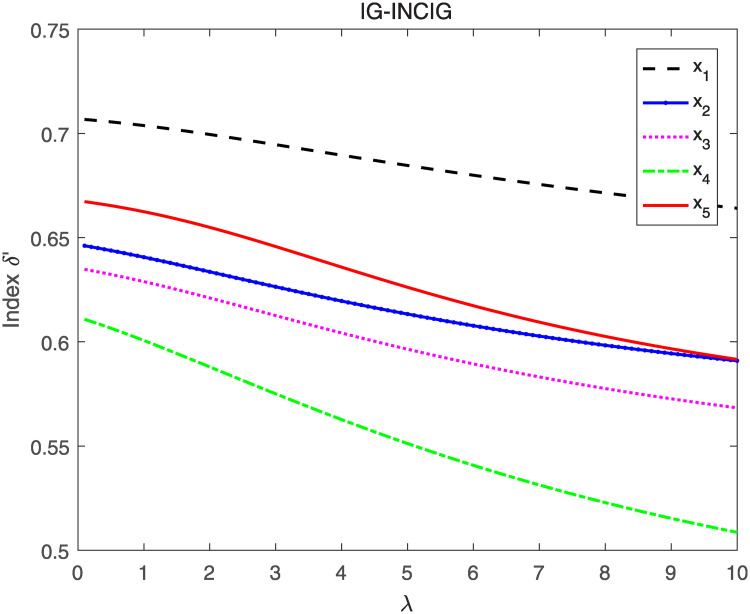
The result when IG-INCIG and index *δ*′ are adopted.

Utilizing the IG-INCIA and IG-INCIG to aggregate interval neutrosophics information respectively and evaluating the aggregated INNs by index *δ* and *δ*′, the ranking results when λ’s value vary from 0 to 10 are shown in Figs 5–8.

As seen in Figs 5 and 6, the ranking of the alternatives changes when the value of λ vary from 0 to 10. The order is *x*_1_ ≻ *x*_5_ ≻ *x*_2_ ≻ *x*_3_ ≻ *x*_4_ when λ is less than 3, and it changes to *x*_1_ ≻ *x*_3_ ≻ *x*_4_ ≻ *x*_5_ ≻ *x*_2_ when λ is greater than 6. The results indicate that the value of λ affects the final order, thus λ is important in the process of information aggregating. In the decision-making, the appropriate parameter should be selected according to the actual situation. Figs 7 and 8 show that the order remains unchanged when IG-INCIG are used to aggregated the interval neutrosophics information. Although the value of the indices varies with the parameter λ, the order of the alternatives is *x*_1_ ≻ *x*_5_ ≻ *x*_2_ ≻ *x*_3_ ≻ *x*_4_. That is to say, the operator IG-INCIG is less sensitive to parameter λ than the operator IG-INCIA. Therefor, when there is no information about the parameter λ, the operator IG-INCIG should be used for interval neutrosophics information aggregation.

What’s more, Fig 8 shows that when λ is greater than a certain value, the alternative *x*_5_ and *x*_2_ are indifferent according to the value of index *δ*′, but that phenomenon does not occur when index *δ* are adopted, which indicate that index *δ* has a better degree of discrimination than index *δ*′ when λ is great enough.

A comparison of the proposed method with some existing MCDM approaches in dealing with the mentioned example is presented. As shown in Table 4, the best alternative is *x*_1_ and the worst alternative is *x*_4_ for all referenced method, although the ranking order is different by using different methods. The results in this study are consistent with the result in existing research when IG-INCIG operator is adopted or IG-INCIA operator is adopted when λ is less than a certain value. But when IG-INCIA operator is adopted and λ is great enough, the worst alternative is *x*_2_. The method in this paper shows that the value of the parameter affects the final sorting result and must be taken seriously. The reason that different methods produce different ranking order is that the method in specific paper is aimed at specific MCDM. For example, the method based on cross entropy proposed in [41] aims to solve the problems which have incomplete weight information. The outranking approach in [43] fits to the situation where the weight of criteria are unknown. The proposed method aims to deal with the situation where the criteria are dependent and the evaluation information is expressed by INNs. The ranking results indicate that the proposed method is effective in dealing with MCDM with interactive attributes.

**Table 4:** The ranking results of different approach.

Approach	Ranking
A method based on cross entropy [41]	*x*_1_ ≻ *x*_2_ ≻ *x*_5_ ≻ *x*_3_ ≻ *x*_4_
An outranking approach [43]	*x*_1_ ≻ *x*_2_ ≻ *x*_5_ ≻ *x*_3_ ≻ *x*_4_
An extended TOPSIS method [39](Hamming distance is adopted)	*x*_1_ ≻ *x*_5_ ≻ *x*_2_ ≻ *x*_3_ ≻ *x*_4_
An extended TOPSIS method [39](Euclidean distance is adopted)	*x*_1_ ≻ *x*_2_ ≻ *x*_5_ ≻ *x*_3_ ≻ *x*_4_
The proposed method(IG-INCIA and index *δ* are adopted, when λ < 3)	*x*_1_ ≻ *x*_5_ ≻ *x*_2_ ≻ *x*_3_ ≻ *x*_4_
The proposed method(IG-INCIA and index *δ* are adopted, when λ > 6)	*x*_1_ ≻ *x*_3_ ≻ *x*_4_ ≻ *x*_5_ ≻ *x*_2_
The proposed method(IG-INCIG and index *δ* are adopted)	*x*_1_ ≻ *x*_5_ ≻ *x*_2_ ≻ *x*_3_ ≻ *x*_4_

## Conclusions

This paper proposes a method for MCDM problems with interactive criteria and interval neutrosophics information. Two aggregation operators are presented to aggregate the evaluation information of criteria. Some properties of the aggregation operators are discussed simultaneously. What’s more, we put forward two indices to rank the aggregated INNs based on geometrical structure of three-dimension space. Then, a MCDM method is proposed based on the aggregation operators and order relations. The decision making process of the proposed method is illustrated by analysing a numerical example. From numerical example analysis, the method we proposed can effectively deal with the situation where the criteria are dependent and the evaluation information is expressed by INNs. Obviously, ranking results indicate that the method is effective in dealing with MCDM with interactive attributes. We argue that the appropriate parameter should be selected according to the actual situation in the process of decision-making. When there is no information about the parameter, the operator IG-INCIG should be used for interval neutrosophics information aggregation. And when λ is great enough, the index *δ* has better performance that index *δ*′. Finally, a comparative analysis of the proposed approach and some existing methods is also conducted to verify the practicality and effectiveness of the method. The research integrates the INNs aggregation operators based on Choquet integral into a unified framework, thus enriching and expanding the theory and methods of MCDM.

Furthermore, some limits are also in our study and need to be improved in future. For instance, large scale data sets may be required during simulations, which can provide sufficient evidences to the empirical conclusion. In addition, Using the proposed MCDM method to solve some practical instances in other areas such as supply chain and human resource may produce better performance.
